# Alteration of LARGE1 abundance in patients and a mouse model of 5q-associated spinal muscular atrophy

**DOI:** 10.1007/s00401-024-02709-x

**Published:** 2024-03-12

**Authors:** Andreas Roos, Linda-Isabell Schmitt, Christina Hansmann, Stefanie Hezel, Schahin Salmanian, Andreas Hentschel, Nancy Meyer, Adela Della Marina, Heike Kölbel, Christoph Kleinschnitz, Ulrike Schara-Schmidt, Markus Leo, Tim Hagenacker

**Affiliations:** 1 Department of Pediatric Neurology, Center for Neuromuscular Disorders, Center for Translational Neuro- and Behavioral Sciences (C-TNBS), University Hospital Essen, Hufelandstr. 55, 45147 Essen, Germany; 2 Division of Neurology, Department of Medicine, The Ottawa Hospital, Brain and Mind Research Institute and Children’s Hospital of Eastern Ontario Research Institute, University of Ottawa, Ottawa, Canada; 3 Department of Neurology, Center for Translational Neuro- and Behavioral Sciences (C-TNBS), University Hospital Essen, Hufelandstr. 55, 45147 Essen, Germany; 4 Leibniz-Institut Für Analytische Wissenschaften—ISAS-e.V., Dortmund, Germany, Otto-Hahn-Strasse 6B, 44227 Dortmund, Germany

**Keywords:** SMA, Proteomics, Spinal cord, Skeletal muscle, CSF, Serum

## Abstract

Spinal muscular atrophy (SMA) is a neuromuscular disorder caused by recessive pathogenic variants affecting the survival of motor neuron (*SMN1*) gene (localized on 5q). In consequence, cells lack expression of the corresponding protein. This pathophysiological condition is clinically associated with motor neuron (MN) degeneration leading to severe muscular atrophy. Additionally, vulnerability of other cellular populations and tissues including skeletal muscle has been demonstrated. Although the therapeutic options for SMA have considerably changed, treatment responses may differ thus underlining the persistent need for validated biomarkers. To address this need and to identify novel marker proteins for SMA, we performed unbiased proteomic profiling on cerebrospinal fluid derived (CSF) from genetically proven SMA type 1–3 cases and afterwards performed ELISA studies on CSF and serum samples to validate the potential of a novel biomarker candidates in both body fluids. To further decipher the pathophysiological impact of this biomarker, immunofluorescence studies were carried out on spinal cord and skeletal muscle derived from a 5q-SMA mouse model. Proteomics revealed increase of LARGE1 in CSF derived from adult patients showing a clinical response upon treatment with nusinersen. Moreover, LARGE1 levels were validated in CSF samples of further SMA patients (type 1–3) by ELISA. These studies also unveiled a distinguishment between groups in improvement of motor skills: adult patients do present with lowered level per se at baseline visit while no elevation upon treatment in the pediatric cohort can be observed. ELISA-based studies of serum samples showed no changes in the pediatric cohort but unraveled elevated level in adult patients responding to future intervention with nusinersen, while non-responders did not show a significant increase. Additional immunofluorescence studies of LARGE1 in MN and skeletal muscle of a SMA type 3 mouse model revealed an increase of LARGE1 during disease progression. Our combined data unraveled LARGE1 as a protein dysregulated in serum and CSF of SMA-patients (and in MN and skeletal muscle of SMA mice) holding the potential to serve as a disease marker for SMA and enabling to differentiate between patients responding and non-responding to therapy with nusinersen.

**Supplementary Information:**

The online version contains supplementary material available at 10.1007/s00401-024-02709-x.

## Introduction

Spinal muscular atrophy (SMA), a neuromuscular disease, is the second most common recessive disorder and caused by pathogenic variants affecting the Survival of Motor Neuron (*SMN1*) gene (localized on the long arm of chromosome 5; 5q-related SMA) subsequently leading to reduced protein abundances [17]. SMA is characterized by motor neuron (MN) degeneration leading to severe muscular atrophy, which in the past was often leading to early death [4]. Depending on the expression of *SMN2*, a homologous gene copy of *SMN1*, the severity of SMA might range from cases with prenatal onset to cases with adult onset. Given that *SMN2* harbors a polymorphism in exon 7, leading to the production of a protein with reduced stability, expression of *SMN2* can only insufficiently compensate the loss of functional SMN1 [19]. Along this line, therapies have mainly focused on increasing full-length SMN expression in patients. Currently, three therapies for SMA are available. These are either based on the promotion of exon-7 inclusion in *SMN2*-derived transcripts using specific Antisense Oligonucleotides [3, 11] or the risdiplam molecule [8], or on AAV-based *SMN1* gene replacement [21]. Although these therapies significantly ameliorate the severity and progression of SMA, they do not constitute a full cure for SMA. Existing proteinogenic biomarkers that enable disease and therapy monitoring are mostly restricted to cerebrospinal fluid (CSF), indicating the need for less invasive markers providing relevant information. Moreover, there is a significant number of non-responders, and an intervention delay strongly impacts on the efficacy—later stages of motor neuron degeneration cannot be reversed by restoration of SMN-expression [6, 11, 14, 22]. Thus, complementary, SMN-independent strategies are crucial and should target such SMN-irreversible degenerative processes. Those are defined as pathological alterations that are not reversed by SMN-restoration for a given dose and intervention delay [14]. On the other hand, strengthening the activation of molecular processes facilitating neuronal survival might reflect an additional starting point for the invention of supportive therapeutic concepts. Furthermore, this again accentuates the need for (prognostic and minimally invasive) biomarkers predicting motor-response to allow for optimal clinical decision-making [27]. In addition to the *SMN2* copy number and less frequent genetic modifiers, only few (invasive) proteinogenic biomarkers are available for SMA, including Cathepsin D [26] and neurofilaments (pNF-L, light-chain; pNF-H, heavy chain), which are intermediate filaments and structural proteins of the neuronal cytoskeleton. Both intermediate filaments are secreted to serum/plasma and CSF during axonal damage, thus serving as non-specific markers of neurodegeneration in different diseases [25, 31]. In the plasma of younger patients with SMA type 1 and 2, pNF-H was found to be increased, and a faster decrease was detected during nusinersen treatment in these patients compared to untreated patients [31]. A recent study applying proteomic profiling of CSF confirmed the suitability of pNF-L and Cathepsin D and moreover introduced Arylsulfatase B (ARSB), Ectonucleoside triphosphate diphosphohydrolase 2 (ENTPD2), and Gamma-interferon-inducible lysosomal thiol reductase (IFI30) as the CSF-proteins most predictive of clinical improvement upon therapeutic intervention with nusinersen [1]. However, it is important to note that significant changes are mostly identified in patients younger than 1 year of age, limiting the use in clinical routine. In accordance, analysis of CSF for neurofilament level in SMA patients undergoing nusinersen treatment likewise showed a decline in a smaller cohort of young type 1 patients [24] without significant correlation with motor response in older patients [9, 29, 30].

In the light of the emerging and yet available treatment options for SMA and the remaining need to define biomarkers [28], we performed a proteomic-based discovery study on CSF derived from SMA type 3 patients before and under nusinersen treatment enabling the identification of LARGE1 as a protein increased in SMA patients pre- and post-treatment with nusinersen compared to controls. Based on this finding, LARGE1 levels were further studied in CSF and serum samples derived from pediatric SMA patients (pre-symptomatic as well as type 1, 2 and 3) and derived from adult SMA type 3 patients by enzyme-linked immunosorbent assay (ELISA). Investigation of serum aimed to define the potential of LARGE1 to serve as a minimal invasive proteinogenic marker. Additionally, LARGE1 levels were studied by immunofluorescence in the spinal cord and skeletal muscle of a murine model of SMA presenting with late disease onset at two different ages.

## Materials and methods

### Cerebrospinal fluid samples of SMA patients

CSF samples of 35 adult patients (age 16–65 years; demographic data of patients are provided in Supplementary Tab. 1 online resource) and of 14 pediatric cases (age 8–12 years; demographic data of patients are provided in Supplementary Tab. 2 online resource) with genetically proven 5q- SMA (type 1, 2, or 3, respectively), as well as controls, were analyzed. Moreover, serum samples derived from 8 adult patients (age 16–65 years) 3 and as well as 8 adult controls were analyzed in addition to 14 pediatric cases (age 1–6 years) with 5q-SMA (SMA type 1 to 3). All patients or their caregivers gave written informed consent. Control CSF was obtained from non-SMA patients (diagnostic procedure to exclude CNS disease; non-disease controls) and from non-SMA patients with inflammatory (*n* = 11 pediatric and *n* = 3 adult) and non-inflammatory CNS diseases (*n* = 6 pediatric and *n* = 2 adult) serving as disease controls. Serum control samples were obtained from healthy donors (non-disease controls) or patients suffering from other neuromuscular conditions (disease controls with genetically confirmed *VWA1*-related neuromyopathy (adult; *n* = 6), *BICD2*-related spinal muscular atrophy with lower extremity predominance (SMALED with adult onset; *n* = 6), and *CHRNE*-related congenital myasthenic syndrome (pediatric; *n* = 7)). SMA-patients responding to therapeutic intervention with nusinersen (were distinguished from such not responding according to the Scores on the Children’s Hospital of Philadelphia Infant Test of Neuromuscular Disorders (CHOP INTEND). Ranging from 0 to 64, with higher scores indicating better motor function; a CHOP INTEND non-response was defined as a failure of an increase of at least 4 points from baseline. The Hammersmith Infant Neurological Examination (HINE) is a neurological examination consisting of 26 items, each scored on a scale of 0 to 3, designed for evaluating infants between 2 and 24 months of age. Motor scores of adult patients were measured using the Hammersmith Functional Motor Scale-Expanded (HFMSE) score or the Revised Upper Limp Module (RULM). Treatment response was classified with an increase of 3 or more motor points. The time point of 180 days post-treatment was chosen as it reflects the ending of the nusinersen “loading phase” and the beginning of the “maintenance phase”, thus providing the full nusinersen effect for each patient and providing robust information regarding the value of therapy markers. Study approval was obtained from the University Duisburg-Essen ethics committee (approval number 18-8285-BO).

### Proteomic profiling on human CSF

#### Sample preparation

Human CSF samples were stored on -80 °C until further processing. A total of 50 μl of each sample (*n* = 16; 2 samples from non-SMA affected individuals, 4 samples from SMA patients not responding to treatment with nusinersen and 10 samples from SMA patients responding to treatment with nusinersen) was denatured with Biognosys’ Denature Buffer. Reduction and alkylation were carried out by adding Biognosys’ Reduction/Alkylation Solution for 1 h at 37 °C. Digestion was then performed with 0.5 μg trypsin (Promega) per sample overnight at 37 °C.

#### Clean-up for mass spectrometry

After digestion, samples were desalted using a BioPureSPEMIDI C18 spin plate (The Nest Group) following the manufacturer’s instructions and dried using a SpeedVac system. Peptides were then dissolved in 20 μl of 1% acetonitrile containing 0.1% formic acid (FA). Prior to mass spectrometric measurements, iRT calibration peptides from Biognosys were added to all samples prepared in this manner. Peptide concentrations were determined using a BCA assay (Thermo Fisher).

#### HPRP fractionation

To perform high-pH fractionation of peptides, equal volumes of digested samples were pooled according to time point (pool 1: baseline, pool 2: 6 months). Ammonium hydroxide was added to achieve a pH > 10. Fractionation was performed using a DionexUltiMate3000 RS pump (ThermoScientific) on an Acquity UPLC CSH C18 1.7 μm, 2.1 × 150 mm column (Waters). The gradient was 1% to 40% solvent B in 20 min, the solvents were A: 20 mM ammonium format in water, B: acetonitrile. Fractions were collected every 30 s and sequentially combined into 6 fraction pools. These were allowed to dry and dissolved in 17 μl solvent A. Before mass spectrometric analyses, they were spiked with iRT calibration peptides from Biognosys. Peptide concentrations were determined using a UV/Vis spectrometer (SPECTROstarNano, BMG Labtech).

#### Shotgun LC–MS/MS for spectral library generation

For DDA LC–MS/MS analyses, 2 µg of peptides per sample or fraction were loaded onto a self-packed C18 column (Dr. Maisch ReproSilPur, 1.9 μm particle size, 120 Å pore size; 75 μm inner diameter, 50 cm length, New Objective) in a Thermo Scientific Easy nLC1200 nano-liquid chromatography system. This system was coupled to a Thermo Scientific Q Exactive mass spectrometer with a standard nano-electrospray source. The following were used as solvents: A: 1% acetonitrile in water containing 0.1% FA; B: 15% water in acetonitrile containing 0.1% FA. The following method was used as the nonlinear gradient: 1–52% solvent B in 120 min, followed by 52–90% B in 10 s, 90% B for 10 min, 90–1% B in 10 s, and 1% B for 5 min. The MS method used was a modified TOP12 method by [15]. Full MS covered the m/z range of 350–1650 with a resolution of 70 000 (AGC target value was set to 3e6) and was followed by MS/MS scans with a resolution of 17 500 (AGC target value was 5e5). The isolation width of the MS/MS acquisition precursor was 2 m/z, while the normalized collision energy was set at 25 (10% staged collision energy) and the default charge state was 2 + .

#### HRM mass spectrometry acquisition

Exactly as for the DDA LC–MS/MS measurements, 2 μg of peptides per sample were loaded onto the in-house packed C18 column in a Thermo Scientific Easy nLC1200 nano-liquid chromatography system for the DIA measurements. The system was coupled to a Thermo Scientific Q Exactive HF mass spectrometer equipped with a standard nano-electrospray source. The LC solvents were A: 1% acetonitrile in water containing 0.1% FA; B: 15% water in acetonitrile containing 0.1% FA. The nonlinear LC gradient was 1–55% solvent B in 120 min, followed by 55–90% B in 10 s, 90% B for 10 min, 90–1% B in 10 s, and 1% B for 5 min. The DIA method included a one full range scan followed by 22 DIA windows.

#### Database search of LC–MS/MS data and spectral library generation

Shotgun and HRM mass spectrometry data were analysed using SpectroMine software (Biognosys). The false discovery rate was set to 1% at the peptide and protein levels. Data were validated against a human UniProt protein database (Homo sapiens, 2019-–07-01). The search preferences were set as follows: 2 missed cleavages were allowed. Modifications were set as variable for N-term acetylation, methionine oxidation, deamidation (NQ), carbamylation (KR)). To create a CSF resource spectral library, shotgun data were obtained from commercially available healthy individual CSF and searched using the above settings. Shotgun, HRM, and resource search data were used to create a hybrid spectral library (library ID: 2452_R03) in SpectroMine software.

#### HRM data analysis

HRM mass spectrometric data were analysed using Spectronaut Pulsar software (Biognosys) with the hybrid spectral library generated in this project. The false discovery rate on protein and peptide level was set to 1%, data was filtered using row-based extraction. The HRM measurements analyzed with Spectronaut were normalized using local regression normalization [2].

#### Data analysis

Data analysis and plotting was performed in R. Distance in heat maps was calculated using the “manhattan” method, the clustering using “ward.D” for both axis. Principal component analysis was conducted in R using prcomp and a modified ggbiplot function for plotting, and partial least squares discriminant analysis was performed using mixOMICS package.

### Enzyme-linked immunosorbent assay (ELISA)

The level of LARGE1 were measured in CSF and serum samples derived from adult and pediatric SMA patients and matching controls by making use of the ELISA technique according to the manufacturer’s protocol (#MBS281249, MyBioScource, San Diego, CA, USA).

### Animals

SMN-deficient mouse model FVB.Cg-*Smn1*^*tm1Hung*^ Tg(SMN2)2Hung/J (Jackson #005058), reflecting later-onset SMA, homozygote for the murine *SMN1* knockout, and the insert of human *SMN2* (4 copies) were purchased from Jackson Laboratory (Bar Habor, ME, USA) and bred in the Animal Research Lab of the University Hospital Essen. Male and female mice were used for spinal cord tissue (P10, P20, P28, P42, and P52) harvesting and muscle tissue (*Musculus tibialis anterior* (TA)) at postnatal days P10, P28, and P52. Age-matched male FVB/N mice (wild-type, wt) served as control. At P42, SMA mice already show loss of spinal motor neurons [18].

All animals were kept on a 12/12 h light/dark cycle with water and standard food pellets available ad libitum.

All experiments were conducted under the animal welfare guidelines of the University Duisburg Essen. Furthermore, the use of the SMA mouse model was approved by the State Agency for Nature, Environment and Consumer Protection (LANUV) in North Rhine-Westphalia (reference number 81–02.04.2020.A335).

### Preparation of murine spinal cord tissue and tibialis anterior as well as human quadriceps muscle slices

Lumbar spinal cord tissue of late-onset SMA and wild-type mice were snap-frozen in liquid nitrogen and stored at − 80 °C until usage. Spinal cord cryosections of 20 µm were prepared. Every fifth section of each spinal cord is placed on one independent microscopy slide.

The murine TA was removed and immediately snap-frozen in a mold of Tissue-Tek^®^ O.C.T.^™^ Compound on dry ice in isopropanol. Afterwards, muscles were stored at − 80 °C until usage. Muscle cryosections of 12 µM were prepared for immunostaining studies. For immunostaining studies on quadriceps muscle derived from pediatric SMA type 3 patients (muscle biopsies were collected for diagnostic purposes), 10 µM cryosections were used.

### Immunostaining of spinal cord tissue slices and muscle biopsy specimen

Murine lumbar spinal cord or TA sections were fixed in 4% Paraformaldehyde, washed, permeabilized (PBS, 0.1 *v/w* Triton X-100), and blocked (PBS, 5% bovine albumin serum). Primary antibodies for motor neurons (anti-SMI-32, mouse, 1:400, #801,701, BioLegends, San Diego, CA, USA), LARGE1 (anti-LARGE1, rabbit, 1:500, #PA5-78,393, Thermo Fisher Scientific, Taufkirchen, Germany), Golgi marker GM130 (anti-GM130, mouse, 1:500, #AB169276, Abcam, Cambridge, United Kingdom) and BiP (GRP78) (anti-BiP BD, mouse, 1:500, Transduction Laboratories #610,978) were diluted in blocking solution and incubated at 4 °C overnight. Sections were washed, and secondary antibodies (goat anti-rabbit, goat anti-mouse, 1:300, Dianova, Hamburg, Germany) and DAPI (1:1000, Sigma-Aldrich, Taufkirchen, Germany) were diluted in blocking solution. Sections were incubated for 1.5 h at room temperature.

Images were obtained using a Zeiss Axio Observer.Z1 Apotome (Zeiss, Jena, Germany) fluorescence microscope and Zeiss Zen software to determine the relative protein levels. All microscope settings such as laser intensity, exposure time, or contrast were kept the same to analyze LARGE1, GM130 and BiP immunoreactivities.

Immunoreactivity of LARGE1 in the entire ventral horn or SMI-32 positive spinal motor neurons as well as in the TA sections and immunoreactivity of GM130 and BiP was measured using Image J software (NIH, Bethesda, MD, USA). LARGE1, GM130 or BiP immunoreactivity was measured and normalized against the background of each slice.

The relative protein level of LARGE1 or GM130 was calculated by normalizing the intensity of SMA tissue against wild-type tissue.

Colocalization of LARGE1 and GM130 as well as LARGE1 and BiP was determined using Image J software plug-in “Coloc 2” and calculation of Pearson coefficient.

### Western blot analysis

We performed Western blot analysis to substantiate the evaluated protein level by immunostaining. Therefore, the spinal cord tissue of SMA or wt mice were homogenized in RIPA buffer containing a protease inhibitor cocktail (Roche, Germany). The amount of protein in those lysates was determined by a bicinchoninic acid (BCA) protein assay.

Ten micrograms of protein were applied to 4–15% TGX Stain-Free gels (Biorad, Germany), and proteins were transferred to 0.2 µm nitrocellulose membranes using a semi-dry blotting technique. Images of membranes were taken for total-protein evaluation. Afterward, the membranes were incubated in fast-blocking solution (Biorad, Germany) under gentle agitation for 10 min at RT. Then, the membranes were incubated with primary antibodies (in blocking solution) against LARGE1 (anti-rabbit, 1:10,000) at 4 °C overnight. Primary antibody against β-actin (hFAB,1:10,000 #12,004,164 BioRad, Germany; anti mouse 1:10,000 #ab8226 Abcam, United Kingdom) as well as GAPDH (anti-mouse, 1:10,000, # MA5-15,738, Thermo-Fisher) was used as an additional loading control.

After washing, the membranes were incubated with anti-rabbit horseradish peroxidase-coupled secondary antibody for 1.5 h at RT. Immunoreactivity was detected using an enhanced chemiluminescence substrate and a Western blot imaging system (Biorad, Germany).

Analysis of Western blot signals was performed using Biorad imaging software. First, the signal of each protein and actin lane was measured. Then, the protein signal of each lane was normalized to its total protein value (PonceauS). Finally, the calculated protein level of SMA mice was further normalized to the value detected in age-matched wt mice, and additionally the wt mice were normalized.

All Western blots and total protein staining used for mean value calculations are provided in the supplementary material (Supplementary Figs. 7–8 online resource).

## Results

### Unbiased proteomic profiling identified increase LARGE1 in CSF derived from SMA type 3 patients responding to therapy with nusinersen

**Fig. 1:**
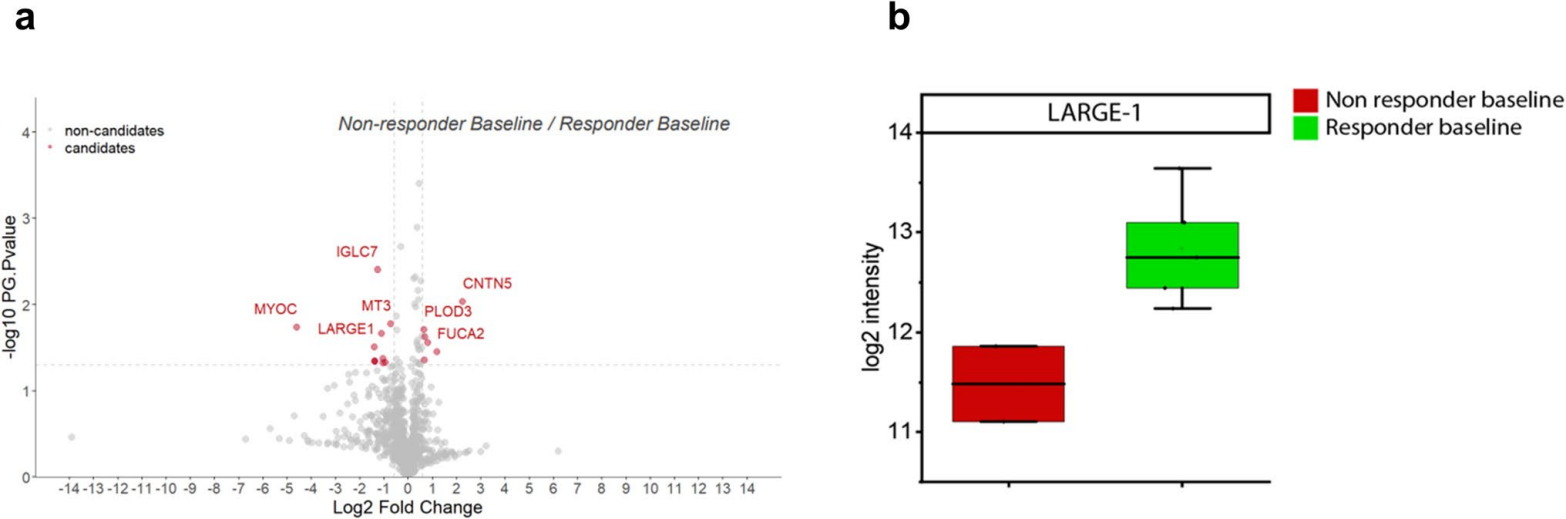
Proteomic profiling of CSF derived from adult SMA patients. **a** Volcano plot of proteomic data obtained by the comparison of the proteinogenic signatures of CSF derived from patients showing a response to the therapeutic intervention with nusinersen versus such without clinical amelioration (non-responders); the group of decreased proteins contains LARGE1. **b** Boxplot-based presentation of diverging LARGE1 level in CSF derived from treatment responders and non-responders. Abbreviations: *CSF* cerebrospinal fluid, *SMA* spinal muscular atrophy

To identify novel CSF-related biomarkers of therapeutical relevance for SMA, untargeted proteomic profiling was carried out on CSF derived from adult SMA type 3 patients (n = 14 with additional 2 non-disease controls) pre- and post-treatment with nusinersen as a discovery approach. For post-treatment conditions day 180 (prior fifth application of nusinersen) was chosen. Our cohort also included two patients not showing a clinical amelioration after 180 days of therapy (non-responders). In total, 17 proteins were significantly changed between baseline samples (day 0) from responder and non-responder SMA patients including LARGE1 (ratio 2.62; Log ratio 1.39; p-ANOVA = 0.03) (Fig. 1; Supplementary Tab. 3 online resource). Given that LARGE1 (acting as a bifunctional glycosyltransferase with both alpha-1,3-xylosyltransferase and beta-1,3-glucuronyltransferase activities) is localized to the ER-Golgi network and other ER-Golgi resident proteins were already unveiled as critical factors for motor neuron survival in SMA [5] and other motor neuron diseases such as ALS [10], were selected this protein as promising candidate for further analyses. This decision was also motivated by the known role of LARGE1 in the genesis of neuromuscular diseases (MIM: 608,840 & MIM:613,154).

### ELISA-based quantification of LARGE1 in SMA-patient derived CSF samples

**Fig. 2:**
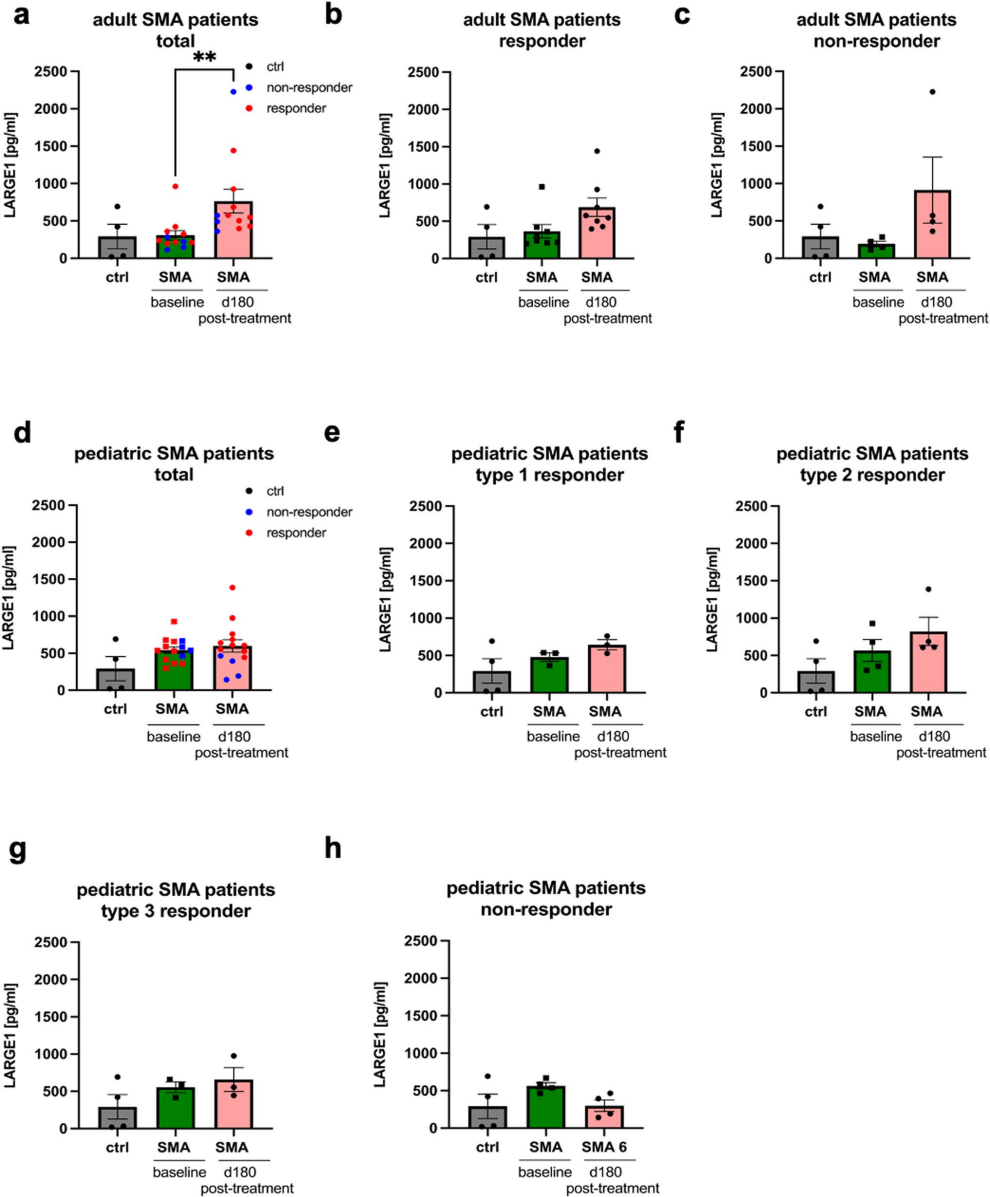
ELISA-based quantification of LARGE1 in CSF samples derived from 5q-associated SMA patients and controls. **a**, **b** and **c** Results obtained in SMA patients with adult onset of disease at baseline visit and 180 days post-treatment with nusinersen. Increase of LARGE1 in the total cohort 180 days post-treatment is statistically significant (*p* < 0.01). **d**–**h** Results obtained in SMA patients with childhood-onset of disease at baseline visit and 180 days post treatment with nusinersen. Abbreviations: *CSF* cerebrospinal fluid, *ctrl* control, *d* day, *SMA* spinal muscular atrophy. *P* values: *p < 0.05, **p < 0.01, or ***p < 0.001

To validate our proteomic findings and to further elucidate the potential of LARGE1 to serve as a (therapeutic relevant) biomarker for SMA, ELISA-based quantification of LARGE1 level was carried out in CSF samples derived from pediatric patients suffering from SMA type 1 (*n* = 3), type 2 (*n* = 4) and type 3 (*n* = 3) responding to therapeutic intervention as well as in 4 pediatric non-responders in addition to adult patients suffering from SMA type 3 (*n* = 18). Moreover, LARGE1 was quantified in pediatric and adult disease controls (inflammatory and non-inflammatory CNS diseases). Results of these studies show an increase of LARGE1 level in CSF derived from adult SMA-patients 180 days post treatment with nusinersen (Fig. 2a and b; *p* < 0.01; *p* = 0.051). This effect was similar in patients responding to treatment and not responding to treatment (Fig. 2b and c). However, in adult patients not responding to therapy, lower LARGE1 levels are identified in CSF at baseline visits compared to controls and patients responding to treatment (Fig. 2b and c). This finding not only confirms our proteomic results but moreover yields into a higher delta value of LARGE1 in pg/ml in the non-responder cohort (720.4) compared to the responder cohort (320.5). In pediatric patients, increase of LARGE1 levels in CSF at baseline visit is more pronounced compared to the observation obtained in the adult cohort and independent of the SMA sub-type or effect of therapeutic intervention with nusinersen. Moreover, a further increase of LARGE1 in CSF is observed in all SMA sub-types responding to therapy, whereas non-responders, in contrast, show a decrease of LARGE1 at day 180 (Fig. 2d–h). The latter finding is in line with our proteomic data. This—in contrast to the data obtained in the adult cohort—in turn results in a negative delta value of LARGE1 in pg/ml in the non-responder cohort (− 256,2) compared to the responder cohort (175,4). LARGE1 levels are significantly increased in pediatric patients compared to adult patients (*p* < 0.05) (Supplementary Fig. 1 online resource). We also quantified the levels of LARGE1 in the CSF of both adult and pediatric patients afflicted with different central nervous system (CNS) diseases, and compared these measurements with those obtained from SMA (Supplementary Fig. 2 online resource). Analysis revealed a significant elevation of LARGE1 in the CSF of SMA patients, especially the pediatric CSF was increased compared to non-inflammatory and inflammatory CSF in pediatric patients (*p* < 0.001; *p* < 0.05). In the pediatric and adult disease controls LARGE1 level were rather decreased: 119.0 pg/ml and 56.4 pg/ml (inflammatory and non-inflammatory CNS diseases in pediatric patients, respectively), as well as 110.7 pg/ml and 45.8 pg/ml (inflammatory and non-inflammatory CNS diseases in adult patients, respectively).

**Fig. 3:**
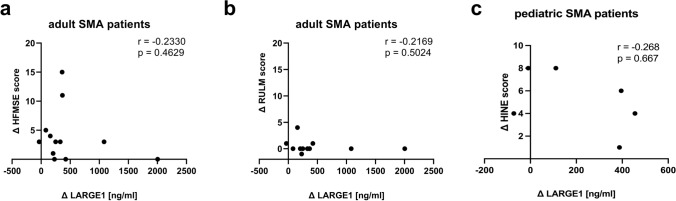
Correlation studies of changes in LARGE1 CSF level in dependence of improvement of motor scores for adult and pediatric SMA patients. **a** HFMSE score in adult patients in correlation with changes of LARGE1 CSF level between baseline visit and day 180 post treatment. **b** RULM score in adult patients in correlation with changes of LARGE1 CSF level between baseline visit and day 180 post treatment. **c** HINE score in pediatric patients in correlation with changes of LARGE1 CSF level between baseline visit and day 180 post treatment. Abbreviations: *CSF* cerebrospinal fluid, *ctrl* control, *d* day, *HINE* Hammersmith infant neurological examination, *HFMSE* Hammersmith functional motor scale—expended, *RULM* revised upper limb module, *SMA* spinal muscular atrophy

To investigate a potential correlation between altered (elevated) LARGE1 levels in CSF and therapeutic outcome in terms of improvement of motor function, delta of different motor scores was calculated for each patient and correlated with delta of LARGE1 protein levels (baseline versus day 180 post treatment). Whereas for RULM, this approach showed no correlation between the increase of LARGE1 in CSF and improved motor function, for HFMSE, four patients showed an increase of LARGE1 without an effect on improved HFMSE score. Thus, eight patients showed increased LARGE1 level accompanied by improvement of the HFMSE score (Fig. 3a and b). HINE score was available for six pediatric patients and showed an increase accompained by increase of LARGE1 level in CSF for four of these patients (Fig. 3c).

### ELISA-based quantification of LARGE1 in SMA-patient derived serum samples

**Fig. 4:**
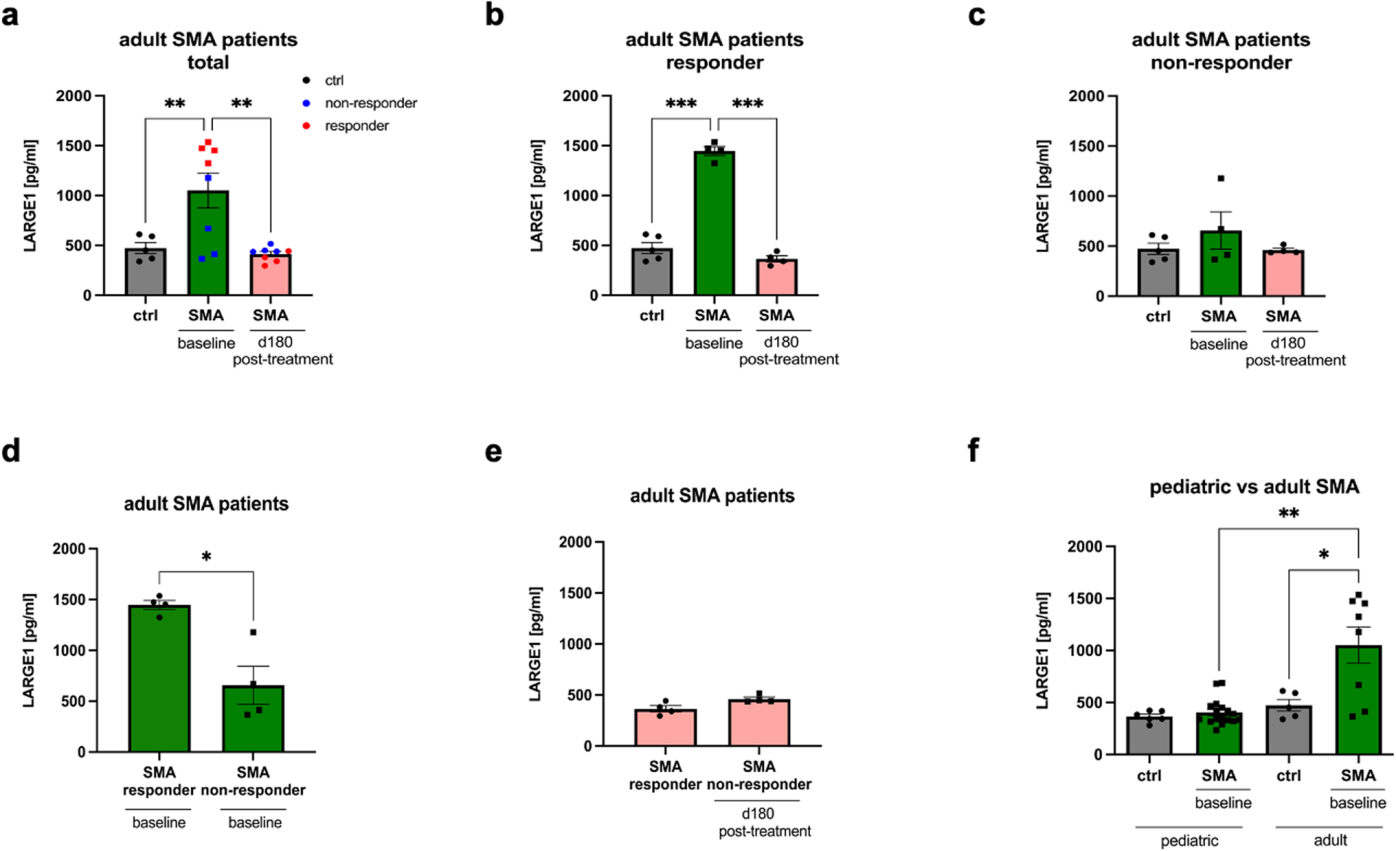
ELISA-based quantification of LARGE1 in serum samples derived from 5q-associated SMA patients and controls. **a**–**c** Results obtained in SMA patients with adult onset of disease at baseline visit. Increase of LARGE1 in the total cohort as well as in the subcohort of responders is statistically significant (*p* < 0.05, *p* < 0.001), respectively. **d** Results obtained in SMA patients with childhood onset of disease at baseline visit. Abbreviations: *ctrl* control, *d* day, *ELISA* enzyme-linked immunosorbent assay, *SMA* spinal muscular atrophy. *P* values: *p < 0.05, **p < 0.01, or ***p < 0.001

We next addressed the potential of LARGE1 to serve as a minimal invasive biomarker by investigating the level in serum samples derived from adult and pediatric SMA patients at baseline visit: the investigation of 8 adult patients (4 responder and 4 non-responder) revealed a significant increase of LARGE1 level in serum samples derived from the patient cohort compared to controls (*n* = 5). However, when comparing responders and non-responders, only patients which show a clinical benefit from therapeutic intervention present with a profound and significant elevation of LARGE1 level whereas the increase in non-responders was rather mild an statistically not significant (Fig. 4a–c). However, in the pediatric cohort (*n* = 17), no increase of LARGE1 serum level was observed in SMA patients (Fig. 4d). A subcategorization of the pediatric cases according to the clinical presentation (pre-symptomatic and SMA 1–3) did also not reveal a statistical significant increase in one of these subgroups (Supplementary Fig. 3 online resource). Furthermore, we conducted a comparative analysis of baseline LARGE1 levels in serum samples derived from pediatric and adult patients with SMA against the serum LARGE1 levels observed in various other neuromuscular disorders (NMD). Serum samples from pediatric and adult SMA patients exhibited significantly elevated LARGE1 levels when compared to those from patients with other NMDs (*p* < 0.05; *p* < 0.01) (Supplementary Fig. 4 online resource). These other NMDs served as disease controls and included *VWA1*-patients (166.4 pg/ml), *BICD2*-patients (164.5 pg/ml), and *CHRNE*-patients (175.4 pg/ml) (Supplementary Fig. 4).

### Mouse model of late-onset SMA shows loss of motor neurons at P42 and P42 but not at P20 or P28

To determine the number of motor neurons in the spinal cord of wild-type or SMA mice, immunostaining for SMI-32, a marker for motor neurons, was performed, and the number of positive neurons was counted (Supplementary Fig. 5a online resource).

At P20 and P28, no reduction in the number of spinal motor neurons in SMA mice was observed, compared to wild-type mice (*p* > 0.05) (Supplementary Fig. 5b, c online resource). This is in contrast to the findings at P42 and P52. We observed a reduction in the number of spinal motor neurons in SMA mice compared to wild-type mice at the same age (P42 and P52) as well as SMA mice at P20 and P28 (Supplementary Fig. 5d, e online resource). The MN loss can be classified into two stages, the active MN loss starting after P28 to P42 and the stagnation phase starting after P42 to P52 (Supplementary Fig. 5f online resource).

### Abundance of LARGE1 in spinal cord tissue of late-onset SMA mouse model is increased at P52 but not at P28

**Fig. 5:**
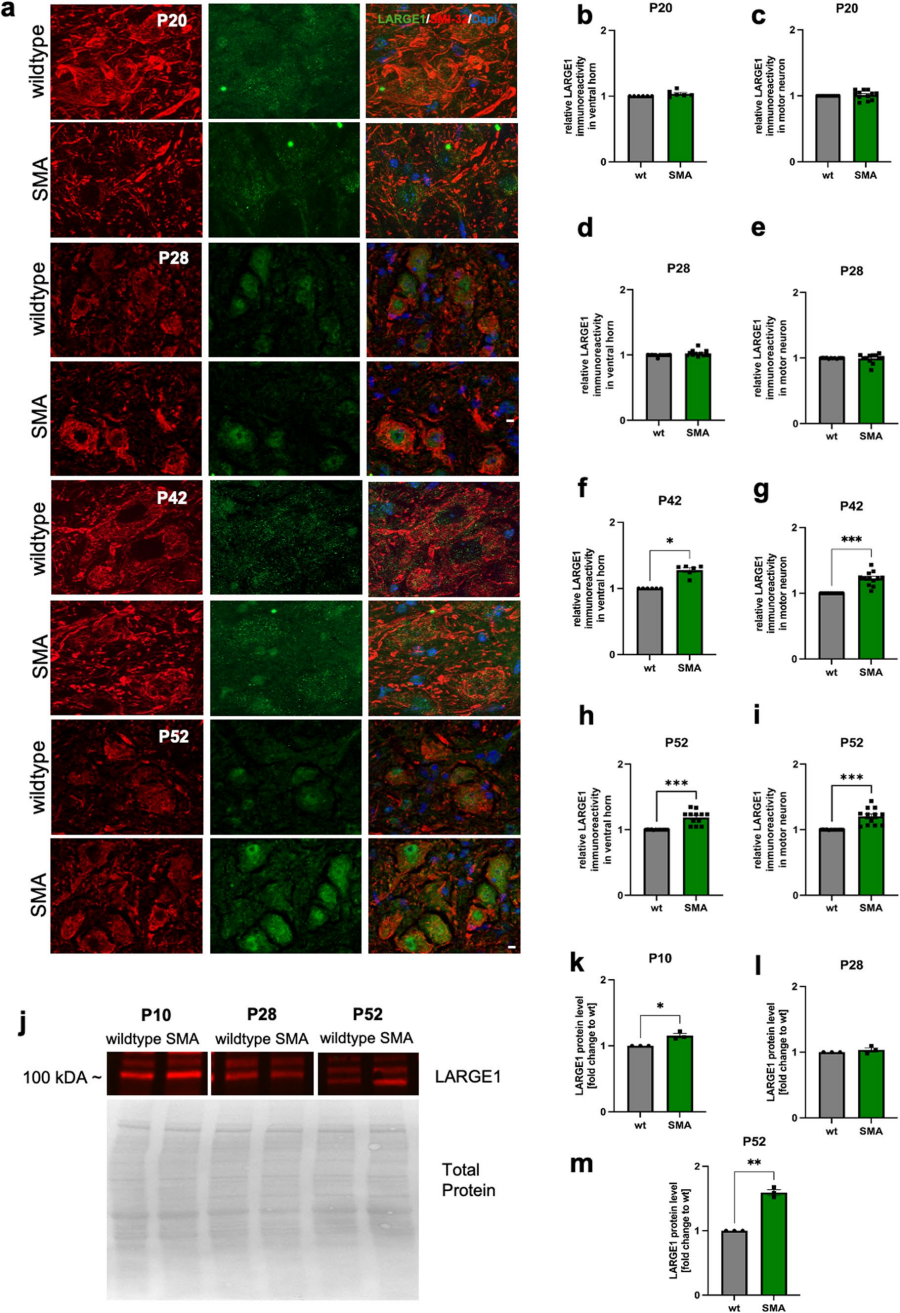
Expression of LARGE1 is increased in a mouse model of late-onset SMA at P52 but not at P28. **a** Immunostaining of spinal cord tissue from wild-type mice (wt) and late-onset SMA mice for LARGE1 (green) and motor neurons (SMI-32, red) at P20, P28, P42 and P52. Nuclei DNA was stained with DAPI (blue). **b**–**e** No differences in LARGE1 immunoreactivity in the spinal cord ventral horn or motor neurons observed between wild-type or SMA mice was (*p* > 0.05). **f**–**i** LARGE1 immunoreactivity was increased in the ventral horn and motor neurons of late-onset SMA mice, compared to wild-type mice (*p* < 0.05; *p* < 0.001). **j** Western blot analysis of LARGE1 protein levels in spinal cord tissue of wt or SMA mice at P10, P28, and P52. Beta-actin was used as a loading control. **k**, **l** SMA mice showed an enhanced LARGE1 protein level at P10 and P52 compared to wt mice (*p* < 0.05; *p* < 0.001). *n* = 3 animals per condition, with 4 slices per animal analyzed (total *n* = 12 slices per condition). Scale bar: 20 µm. Abbreviations: *DNA* deoxyribonucleic acid, *SMA* spinal muscular atrophy, *P* postnatal day, *wt* wild type. *P* values: *p < 0.05, **p < 0.01, or ***p < 0.001

To examine LARGE1 expression in the spinal cord derived from a mouse model of late-onset SMA at two different time points, spinal cord slices were prepared, and immunostaining for LARGE1 and SMI-32 was performed (Fig. 5a). In addition, immunoreactivity of LARGE1 was measured for the entire ventral horn of the spinal cord and within SMI-32 positive neurons as well.

No differences in LARGE1 immunoreactivity between wild-type and SMA mice in spinal cords ventral horn or SMI-32 positive cells were observed at P20 and P28 (*p* > 0.05) (Fig. 5b–e). In contrast, LARGE1 immunoreactivity was increased in the ventral horn (p < 0.05; p < 0.001) and in SMI-32-positive cells (*p* < 0.001) of the late-onset SMA mouse model at P42 and P52 (Fig. 5f–i). Western blot analysis shows an increase of LARGE1 protein levels at P10 (p < 0.05) and P52 (*p* < 0.01) compared to wt mice. No difference occurred at P28 (Fig. 5j–m; Supplementary Fig. 9 online resource).

When comparing LARGE1 immunostaining between SMA-animals and matching controls in relation to the MN loss, identical phases can be identified: a phase of MN loss, commences at P28 and extends until P42, followed by a stagnation phase from P42 to P52. Between P42 and P52, no significant changes in protein levels can be observed (Supplementary Fig. 6a, b online resource). Our Western Blot data show an elevation in LARGE1-protein levels at P10 and P52. When evaluating the temporal progression, LARGE1 exhibits a transient increase at P10, subsequently returning to control levels at P28, and rising again until P52 (Supplementary Fig. 6c and d online resource). Generally, a fundamentally heightened level of LARGE1 protein is evident at P10 (Supplementary Fig. 6c and e online resource). P28 and P52 demonstrate a distinctly reduced protein level of LARGE1 (both wildtype and SMA mouse models) compared to P10 (Supplementary Fig. 4c and e online resource). Further analysis of the non-normalized values from the Western Blot reveals that SMA animals exhibit a significantly elevated protein level at P10 compared to P28 and P52. However, P52 also displays a significant increase relative to P28 (Supplementary Fig. 6e online resource).

### GM130 abundance is increased in motor neurons of late-onset SMA mouse model at P52 without alteration in co-localization with LARGE1

Assuming the relevance of the Golgi-resident glycosyltransferase LARGE1 (https://www.uniprot.org/uniprot/O95461) in the central nervous system pathology of SMA, we next performed immunostaining of spinal cord slices to determine the abundance and distribution within the Golgi. For that purpose, co-staining with Golgi matric protein 130 (GM130, a well-established Golgi marker, was carried out in spinal motor neurons of late-onset SMA mouse model at P28 and P52 (Supplementary Fig. 7a online resource): immunoreactivity of GM130 was measured, and co-localization with LARGE1 was calculated using the Pearson coefficient.

Although we observed increased GM130 immunoreactivity in spinal motor neurons of late-onset SMA tissue at P52 (*p* < 0.001) but not a P28 (*p* > 0.05), compared to wild-type conditions (Supplementary Fig. 3b and d), no alteration in GM130/LARGE1 co-localization was observed between wild-type and late-onset SMA mice at P28 or P52 was observed (*p* > 0.05) (Supplementary Fig. 7c and e online resource).

### BiP abundance is not altered in the late-onset SMA mouse model, but co-localization with LARGE1 is reduced at P52

To further elucidate the impact of subcellular LARGE1 distribution (not co-localizing with GM130) in the etiology of SMA, we moreover performed immunostaining focusing on the localization of LARGE1 to the Endoplasmic Reticulum (ER) forming a functional network with the Golgi. For that purpose, binding immunoglobulin protein (BiP) was selected as a well-established ER marker, and the level of BiP along with its co-localization with LARGE1 were determined in spinal motor neurons of late-onset SMA mouse model at P28 and P52 (Supplementary Fig. 8a online resource): immunoreactivity of BiP was measured, and co-localization with LARGE1 was calculated using the Pearson coefficient.

We observed no changes within the BiP immunoreactivity in spinal motor neurons of late-onset SMA tissue neither at P28 (*p* > 0.05) nor P52 (*p* > 0.05), compared to wild-type conditions (Supplementary Fig. 4b and d). However, the co-localization of BiP and LARGE1 was significantly reduced between wild-type and late-onset SMA mice at P52 (*p* < 0.001) but not at P28 (*p* > 0.05) (Supplementary Fig. 8c and e online resource).

### LARGE1 abundance in tissue of tibialis anterior of late-onset SMA mice is increased at P52 but not at P28

**Fig. 6:**
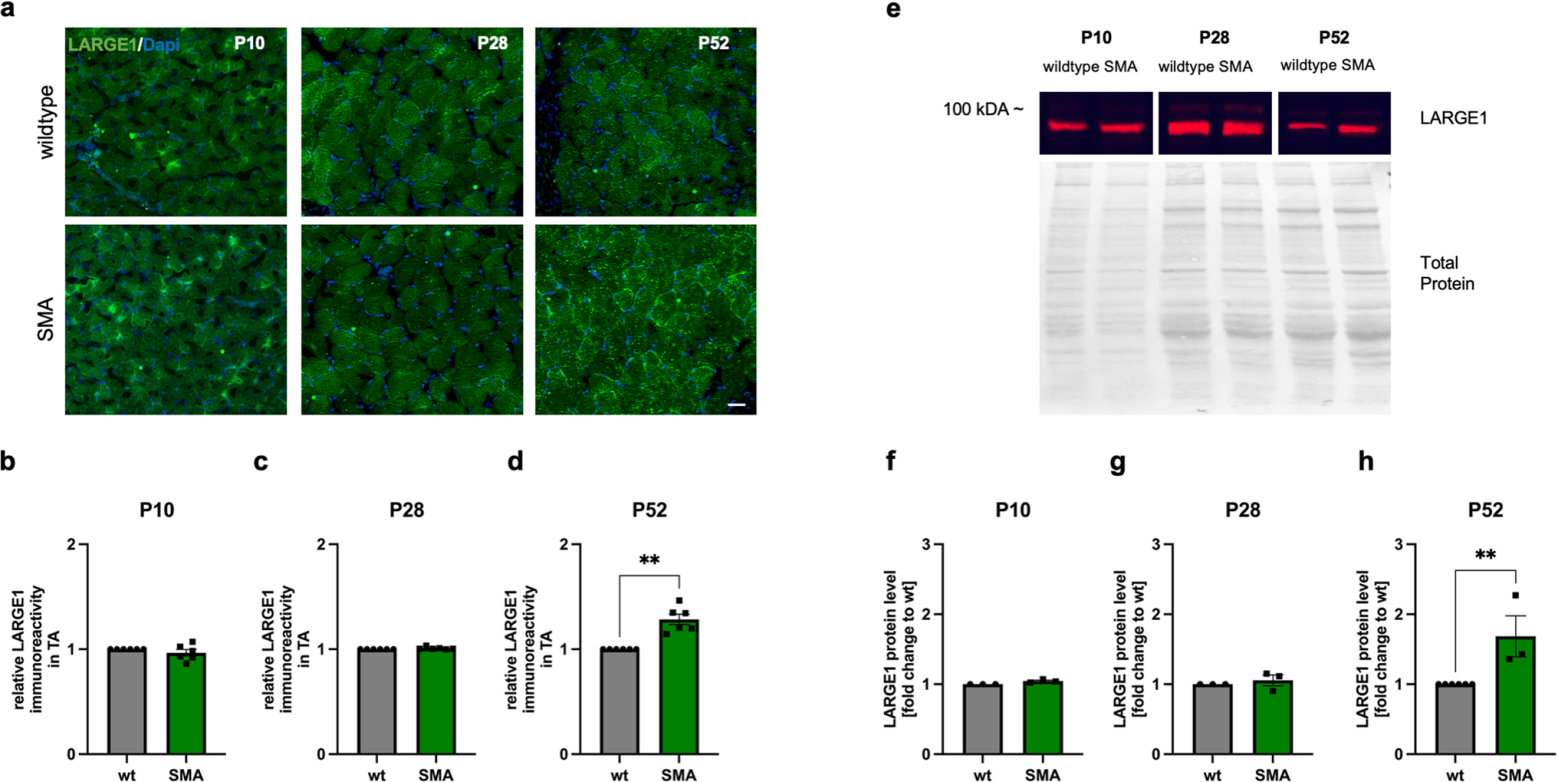
LARGE1 abundance is increased tibialis anterior (TA) muscle in a mouse model of late-onset SMA at P52 but not at P28 or P10. **a** Immunostaining of TA muscle of wild-type (wt) and late-onset SMA mice for LARGE1 (green) at P10, P28 and P52. Nuclei DNA were stained with DAPI (blue). **b**,**c**,**d** Immunoreactivity of LARGE1 in TA muscle was not affected at P10 and P28 (*p* > 0.05), while it was increased in late-onset SMA muscle tissue at P52 (*p* < 0.01). **e** Western blot analysis of LARGE1 protein levels in TA of wt or SMA mice at P10, P28, and P52. **f**, **g**, **h** SMA mice showed an enhanced LARGE1 protein level at P52 compared to wt mice (*p* < 0.01) but no changes were observed at P10 and P28 (*p* > 0.05). *n* = 3 animals per condition, with 2 slices per animal analyzed (total *n* = 6 slices per condition). Scale bar: 20 µm. Abbreviations: *DNA* deoxyribonucleic acid, *P* postnatal day, *SMA* spinal muscular atrophy, *TA* tibialis anterior, *w*t wild type. *P* values: *p < 0.05, **p < 0.01, or ***p < 0.001

To examine LARGE1 level in TA muscle tissues from a mouse model of late-onset SMA at two different time points, cryosections were prepared, and immunostaining for LARGE1 was performed (Fig. 6a).

No differences in LARGE1 immunoreactivity between wild-type and SMA mice at P10 and P28 (p > 0.05) was observed (Fig. 6b and c). In contrast, LARGE1 immunoreactivity was increased in the TA sections of the late-onset SMA mouse model at P52 (*p* < 0.001) (Fig. 6c). Western blot analysis shows an increase of LARGE1 protein levels at P52 (*p* < 0.01) compared to wt mice (Fig. 6e and h; Supplementary Fig. 10 online resource). No difference occurred at P10 and P28 (*p* > 0.05) (Fig. 5e–g; Supplementary Fig. 10 online resource).

## Discussion

Combined results of our study unveil LARGE1 as a novel fluid biomarker in 5q-realted SMA: we demonstrate that LARGE1 is increased in CSF derived from pediatric patients suffering from SMA irrespective of the clinical subtype of the disease but not in CSF derived from children suffering from inflammatory or non-inflammatory CNS diseases. Moreover, in pediatric patients, LARGE1 level further increased in CSF 180 days post-successful treatment with nusinersen whereas an opposite effect was identified in non-responders. These findings may indicate the potential of LARGE1 to serve as a general CSF biomarker in addition to a therapy marker in pediatric SMA. However, increase of CSF LARGE1 in pediatric responders is statistically not significant. Thus, analyses of larger cohorts of treatment responder and non-responder are needed to address the question if this promising finding will yield into statistical significance and will consequently enable a robust patient stratification. Adult SMA patients show increased LARGE1 levels also in patients not responding to therapeutic intervention with nusinersen after 180 days. However, in contrast with the results obtained in the pediatric cohort, adult non-responders show decreased LARGE1 levels compared to controls at baseline visits. In consequence, the fact that the fold of CSF LARGE1-increase upon treatment (almost statistically significant in responders; *p* value = 0.051) does not significantly differ between adult therapy responder and non-responder supports the concept that LARGE1 baseline level should be taking into consideration as a stratification determinant. Regarding the disease genesis and assuming that the increased LARGE1 levels in the serum are attributed to the loss of motor neurons, this molecular observation might reflect that an inadequate therapeutic response is consistent with a greater loss of motor neurons and thus less release of LARGE1 into the CSF. Along this line, we assume that the molecular differences between pediatric and adult SMA-patients reflect the profound motor neuron-degeneration associated with early onset of SMA in the pediatric cohort versus the adult cohort. This assumption is supported by the results of our experiments towards LARGE1 quantification in spinal cord derived from a late-onset SMA mouse model. Here, immunological-based studies unveiled no LARGE1 increase in ventral horn or SMI-32 positive cells at P20 and P28 but at P42 and P52 in turn suggesting a later involvement of LARGE1 increase in the molecular etiology of late-onset SMA. Along this line, the decrease in pediatric non-responders may reflect the progressive loss of LARGE1-expressing neurons impacting on the amount of release of this protein from damaged/ apoptotic neurons to the CSF whereas lower base-line level in adult SMA patients classified as therapy non-responders may reflect an insufficient compensatory LARGE1-upregulation during in the molecular etiology of the disease. This is in line with the assumption that in adult SMA-patients (therapy responders) LARGE1 acts as a possible rescue mechanism (preventing motor neuron degeneration and associated release of cellular proteins to the CSF) and as such is not being released into the CSF like in pediatric patients. A general protective role of LARGE1 in SMA is also suggested by the identification of decreased level in CSF derived from pediatric and adult disease controls in turn supporting the concept a less pronounced LARGE1 degradation in SMA. The weak correlation coefficient between Δscore of each motor function scale and ΔLARGE1 CSF level might indicate that changes in LARGE1 level molecularly precede associated functional outcomes. To prove this assumption further longitudinal studies are needed. Of note, similar observations were described for other SMA-related biomarker studies: a biomarker study on 16 adults with SMA type 3 and 4 under Nusinersen treatment over 22 months reported on a significant decrease of pNF-H and increase of Chitotriosidase-1 (CHIT1) in CSF upon treatment but no correlation of this molecular change with clinical outcome measures. In contrast, a decrease of Chitinase-3-like protein 1 (YKL-40) which was observed upon therapeutic intervention strongly correlated with improvements in the revised upper limb module (RULM) [7]. These findings highlight that therapy markers in SMA do not necessarily correlate with clinical outcome measures even in adult patients in which uniformed standardized motor tests are applicable. Although further studies on larger cohorts are also needed to decipher whether decreased LARGE1 levels at baseline in adult non-responders and decrease of LARGE1 at day 180 after treatment in pediatric non-responders hold the potential to distinguish between patients showing a benefit of nusinersen treatment and such showing no improvement in motor scores, our data suggest that LARGE1 may serve as a therapy relevant marker in CSF of both adult and pediatric patients. The observed LARGE1 decrease in CSF samples derived from pediatric controls hint towards a specificity of LARGE1 increase in CSF in SMA with pediatric onset. However, further studies on CSF samples derived from children suffering from other neurological diseases are needed to draw final conclusions.

Testing the potential of LARGE1 to serve as a minimal-invasive fluid biomarker was addressed by investigating serum samples derived from adult and pediatric SMA patients at baseline visits. Indeed, in adult patients, a significant LARGE1 increase was detected, and subcategorization of patients into responder and non-responder even unraveled a prediction of future clinical response to therapeutic intervention by showing elevated levels only in responders but not in non-responders. However, our data also indicate that in serum, LARGE1 only holds the potential to serve as a predictive biomarker in adult patients, as in pediatric cases, no changes in LARGE1 serum concentration were detected. LARGE1 was also significantly decreased in serum samples derived from disease controls reflecting other neuromuscular diseases with impaired neuromuscular transmission compared to the level detected in SMA-patients. This result is in line with the molecular observation obtained in CSF samples and in principle supports the assumption of a generalized protective role of LARGE1 in SMA (see above). Doubtless, studies on larger cohorts are needed to further validate this finding and thus to introduce LARGE1 as a robust predictive and specific blood biomarker in adult SMA patients. The molecular observation that LARGE1 is not elevated serum of pediatric cases but is higher in adults after a long period of disease progression, might hint toward a constant release of this protein from the muscle cells, which are increasingly vulnerable due to disease progression in adults, whereas in the childhood forms skeletal muscle shows profound damage very early on [26]. This in turn suggests the above-mentioned compensatory role of the LARGE1 increase also in muscle. Functional studies ideally on mouse models representing the different onset forms of SMA are crucial to address the exact role of LARGE1 increase in different tissues in relation to varying severity grades of this neurological disorder. Such studies may also clarify why LARGE1 is conversely elevated with treatment in CSF and along this line if LARGE1 plays different roles within different affected cellular populations.

LARGE1 encodes the bifunctional glycosyltransferase with both alpha-1,3-xylosyltransferase and beta-1,3-glucuronyltransferase activities involved in the maturation of alpha-dystroglycan (DAG1), in turn enabling DAG1 to bind ligands, including laminin 211 and neurexin. Recessive pathogenic variants in *LARGE1* lead to muscular dystrophy-dystroglycanopathy (congenital with brain and eye anomalies type A6; MIM: 613,154) and muscular dystrophy-dystroglycanopathy (congenital with mental retardation type B6; MIM: 608,840). An impact of LARGE1 in proper function of neuromuscular transmission has already been shown in previous studies: deficits in nerve conduction and neuromuscular transmission were observed in LARGE (*myd*) animals, and these deficits were fully rescued by muscle-specific expression of LARGE1, which resulted in restored structure of the neuromuscular junction (NMJ) [13]. Based on this observation, Gumerson and colleagues postulate that in addition to muscle degeneration, impaired neuromuscular transmission contributes to muscle weakness in *myd* mice and that the noted defects are primarily due to the effects of LARGE1 and glycosylated dystroglycan in stabilizing the NMJ [13]. Additionally, a study was designed to explore differentially expressed genes associated with acute and chronic stages of spinal cord injury and unraveled *Large1* as being differentially expressed [23]. Moreover, neuropathological findings indicate that LARGE1 also plays a role in the spinal cord: the spinal cord of a LARGE1-autopsy case was smaller than normal and had a “sombrero hat” configuration, indicative of degeneration or absence of the corticospinal tracts [20]. Our finding of increased LARGE1 in the skeletal muscle of SMA mice accords with its muscular role in proper neuromuscular transmission and moreover supports the concept of SMA being a multisystemic disease with primary vulnerability of skeletal musculature rather than being “a pure” MN-disorder [12]. The impact of elevated LARGE1 in SMA-diseased skeletal muscle derived from a mouse model of SMA type 3 is even supported by the identification of increased LARGE1 serum level in adult SMA patients. However, further functional studies are needed to precisely delineate to role of LARGE1 in muscle- of SMA and, along this line, to link elevated serum levels to muscle cell vulnerability. The finding of increased abundance of LARGE1 in MN of 52-day-old SMA mice supports the concept of a profound role of this protein in MN survival as already indicated by the neuropathological findings obtained in a LARGE1-autopsy case [20]. This assumption is also supported by our finding of increased LARGE1 level in CSF of SMA patients compared to controls characterized by a further LARGE1-increase in pediatric and adult cases under nusinersen therapy. The latter observation might arise from the activation of survival programs in MNs promoted by the restoration of efficient expression of a functional SMA protein. One might speculate that LARGE1 level in CSF allows to differentiate between pediatric responders and non-responders to nusinersen therapy but not between the same ones in adult patients. Again, further studies on larger cohorts of pediatric and adult responders and non-responders are crucial to finally draw this conclusion. GM130 (also known as Golgi subfamily A member 2) is a 130 kDa *cis*-Golgi protein encoded by *GOLGA2*. GM130 is a peripheral membrane component of the cis-Golgi stack that acts as a membrane skeleton maintaining the structure and integrity of the Golgi apparatus and as a vesicle tether that facilitates vesicle fusion to the Golgi membrane and, moreover, acts in cell proliferation and autophagy. Recessive loss of function variants in *GOLGA2* was linked to a neurological phenotype defined by microcephaly, seizures, and myopathy [16], in turn indicating the impact of GM130 on proper integrity and function of both the nervous system and skeletal muscle. Thus, an increase of GM130 in MNs of the SMA mouse model and muscle cells of SMA patients might accord with the activation of defense mechanisms toward cellular survival. However, further functional studies are crucial to confirm the assumption and to precisely delineate the role of GM130 in survival of cell populations along the neuromuscular axis. Given that elevated LARGE1 level did not correlate with an increase of co-localization with GM130, we assumed an interplay of LARGE1 elevation and the ER forming a functional continuum with the Golgi apparatus. To prove this assumption, co-localization studies with BiP (known ER marker) were carried out in murine spinal cord, revealing a decrease in the co-localization of both proteins. This microscopic finding may hint toward an impact of subcellular LARGE1 distribution and further functions of LARGE1 in the molecular etiology of SMA. Further functional studies, such as investigations of the LARGE1 interactome, would be helpful in deciphering the exact role in the pathophysiology of SMA.

## Conclusions

LARGE1 level in CSF enable to distinguish between SMA-cases responding and not responding to nusinersen therapy in both, adult patients (based on baseline level) and pediatric patients (based on level 180 days post-treatment). Moreover, LARGE1 serves as a (minimal invasive) serum biomarker only in adult SMA patients and here even enables to predict treatment response based on baseline protein level.

LARGE1 is increased in spinal cord and skeletal muscle of a SMA3 mouse model upon disease progression. The decreased interaction of LARGE1 with BiP in murine spinal cord and may hint toward a yet undiscovered role of subcellular LARGE1 distribution in the molecular etiology of SMA as a multisystemic disorder. The observed decrease of LARGE1 in CSF and serum derived from patients suffering from other neurological diseases support the concept of a protective role of LARGE1 in SMA. Our combined data thus introduce LARGE1 as a novel SMA biomarker holding the potential to address different demands such as suitability in patient stratification, specificity, pathophysiological relevance and reflection of treatment response.

### Supplementary Information

Below is the link to the electronic supplementary material.

Supplementary file1 (DOCX 31556 KB)

## Data Availability

The data supporting the study findings are available on request from the corresponding author.

## Declarations

### Research involving human and/or animal participants

This study was conducted under the animal welfare guidelines of the University Duisburg Essen, Germany. Furthermore, the use of the SMA mouse model was approved by the State Agency for Nature, Environment and Consumer Protection (LANUV) in North Rhine-Westphalia (reference number 81.02.04.2020.A335).
