# Supplementary material for: Alteration of LARGE1 abundance in patients and a mouse model of 5q-associated spinal muscular atrophy
Source: Acta Neuropathol. 2024 Mar 12;147(1):53. doi: 10.1007/s00401-024-02709-x (PMC10933199; doi:10.1007/s00401-024-02709-x)
Supplement: Supplementary file 1 — Supplementary file1 (DOCX 31556 KB) [file 401_2024_2709_MOESM1_ESM.docx]

Alteration of LARGE1 abundance in patients and a mouse model of 5q-associated spinal muscular atrophy

Andreas Roos^1,2,3,†^, Linda-Isabell Schmitt^4,†,^*, Christina Hansmann^4^, Stefanie Hezel^4^, Schahin Salmanian^4^, Andreas Hentschel^5^, Nancy Meyer ^1^, Adela Della Marina^1^, Heike Kölbel^1^, Christoph Kleinschnitz^4^, Ulrike Schara-Schmidt^1^, Markus Leo^4^ and Tim Hagenacker^4^

^1^ Department of Pediatric Neurology, Center for Neuromuscular Disorders, Center for Translational Neuro- and Behavioral Sciences (C-TNBS), University Hospital Essen, Hufelandstr. 55, 45147 Essen, Germany; [Andreas.Roos@UK-Essen.de](mailto:Andreas.Roos@UK-Essen.de) (A.R.); [Ulrike.Schara-Schmidt@UK-Essen.de](mailto:Ulrike.Schara-Schmidt@UK-Essen.de) (U.S.-S.); [Nancy.Meyer@UK-Essen.de](mailto:Nancy.Meyer@UK-Essen.de) (N.M); [Adela.Dellamarina@UK-Essen.de](mailto:Adela.Dellamarina@UK-Essen.de) (A.M.); [Heike.Koelbel@UK-Essen.de](mailto:Heike.Koelbel@UK-Essen.de) (H.K.)

^2^ Department of Neurology, Heimer Institute for Muscle Research, University Hospital Bergmannsheil, Ruhr-University Bochum, 44789 Bochum, Germany; [Andreas.Roos@UK-Essen.de](mailto:Andreas.Roos@UK-Essen.de) (A.R.)

^3^ Division of Neurology, Department of Medicine, The Ottawa Hospital, Brain and Mind Research Institute and Children's Hospital of Eastern Ontario Research Institute, University of Ottawa, Ottawa, Canada; [Andreas.Roos@UK-Essen.de](mailto:Andreas.Roos@UK-Essen.de) (A.R.)

^4^ Department of Neurology, Center for Translational Neuro- and Behavioral Sciences (C-TNBS), University Hospital Essen, Hufelandstr. 55, 45147 Essen, Germany; [Linda-Isabell.Schmitt@UK-Essen.de](mailto:Linda-Isabell.Schmitt@UK-Essen.de) (L.-I.S.); [Markus.Leo@UK-Essen.de](mailto:Markus.Leo@UK-Essen.de) (M.L.); [Christina.Hansmann@UK-Essen.de](mailto:Christina.Hansmann@UK-Essen.de) (C.H.); [Stefanie.Hezel@UK-Essen.de](mailto:Stefanie.Hezel@UK-Essen.de) (S.H.); [Schahin.Salmanian@stud.uni-due.de](mailto:Schahin.Salmanian@stud.uni-due.de) (S.S.); [Christoph.Kleinschnitz@UK-Essen.de](mailto:Christoph.Kleinschnitz@UK-Essen.de) (C.K.); [Tim.Hagenacker@UK-Essen.de](mailto:Tim.Hagenacker@UK-Essen.de) (T.H.)

^5^ Leibniz-Institut für Analytische Wissenschaften - ISAS - e.V., Dortmund, Germany, Otto-Hahn-Strasse 6B, 44227 Dortmund, Germany; [Andreas.Hentschel@isas.de](mailto:Andreas.Hentschel@isas.de) (A.H.).

***** Correspondence: Linda-Isabell.Schmitt@UK-Essen.de; Tel.: +49 201 723 82366

† these authors contribute equally to the manuscript

**Supplementary Table 1 shows demographic data of included adult SMA patients**

**Table 1:** Demographic data of included adult SMA patients.

| **Age at treatment [years]** | **Sex** | ***SMN2***  **copies** | **SMA**  **subtype** | **Responder/Non-responder** | **Baseline**  **HFMSE score** | **Baseline**  **RULM score** |
| --- | --- | --- | --- | --- | --- | --- |
| 41 | male | 4 | III | Responder | 53 | 37 |
| 18 | male | 3 | III | Responder | 53 | 35 |
| 38 | male | 4 | III | Responder | 44 | 34 |
| 49 | male | 3 | III | Responder | 16 | 25 |
| 22 | male | 4 | III | Responder | 45 | 31 |
| 29 | female | 4 | III | Responder | 57 | 37 |
| 38 | male | 4 | III | Responder | 44 | 34 |
| 28 | male | 3 | II | Responder | 4 | 14 |
| 43 | female | 4 | III | Non-responder | 51 | 37 |
| 46 | male | 4 | III | Non-responder | 30 | 22 |
| 61 | female | 4 | III | Non-responder | 17 | 37 |
| 55 | male | 4 | III | Non-responder | 10 | 21 |
| 29 | male | 4 | II | Non-responder | 0 | 0 |

**Supplementary Table 2 shows demographic data of included pediatric SMA patients**

**Table 2:** Demographic data of included pediatric SMA patients.

| **Age at treatment [months (m)/**  **Years (y)]** | **Sex** | ***SMN2***  **copies** | **SMA**  **subtype** | **Responder/**  **Non-responder** | **Baseline**  **CHOP-Intent** | **Baseline**  **HINE score** | **Baseline HFMSE score** | **Baseline**  **RULM**  **Score** |
| --- | --- | --- | --- | --- | --- | --- | --- | --- |
| 8m | male | 2 | I | Responder | 46 | 2 | N/A | N/A |
| 10m | female | 3 | I | Responder | 32 | 4 | N/A | N/A |
| 12m | female | 3 | I | Responder | 60 | 10 | N/A | N/A |
| 12m | female | 3 | I | Responder | 40 | 5 | N/A | N/A |
| 2y | female | 3 | I | Responder | 32 | 4 | N/A | N/A |
| 6y | male | 4 | II | Responder | N/A | 5 | 5 | 14 |
| 2y | female | 3 | II | Responder | 64 | 17 | 29 | N/A |
| 4y | male | 3 | II | Non-responder | N/A | 14 | 20 | 23 |
| 7y | male | 3 | II | Non-responder | N/A | 12 | 8 | 10 |
| 1m | male | 2 | I | Responder | N/A | 1 | N/A | N/A |
| 5y | female | 4 | III | Non-responder | N/A | 17 | 51 | 43 |
| 6y | male | 4 | III | Responder | N/A | 26 | 60 | 63 |
| 12y | male | 4 | III | Non-responder | N/A | 26 | 63 | N/A |
| 12m | female | 3 | I | Non-responder | 32 | 1 | 10 | N/A |
| 2y | male | 2 | 1 | Non-responder | N/A | 2 | 25 | N/A |
| 3y | female | 2 | III | Responder | N/A | 26 | 58 | 35 |
| 10y | male | 3 | II | Responder | N/A | 9 | 4 | 13 |

**Supplementary Table 3 shows differentially expressed proteins in proteomic analysis of adult SMA patients**

**Table 3:** List of the 17 proteins significantly dysregulated in CSF derived from SMA therapy-responders versus non-responders.

| **Accession** | **Gene ID** | **Protein Description** | **Log ratio** | **Ratio** | **p-value** |
| --- | --- | --- | --- | --- | --- |
| Q99972 | MYOC | Myocilin | 4.61 | 24.44 | 0.02 |
| O95461 | LARGE1 | LARGE xylosyl- and glucuronyltransferase 1 | 1.39 | 2.62 | 0.03 |
| A0A0B4J1X5 | IGHV3-74 | Immunoglobulin heavy variable 3-74 | 1.38 | 2.61 | 0.05 |
| P01860 | IGHG3 | Immunoglobulin heavy constant gamma 3 | 1.38 | 2.61 | 0.05 |
| P0DP04 | IGHV3-43D | Immunoglobulin heavy variable 3-43D | 1.38 | 2.61 | 0.05 |
| A0M8Q6 | IGLC7 | Immunoglobulin lambda constant 7 | 1.26 | 2.39 | 0.00 |
| P81172 | HAMP | Hepcidin | 1.10 | 2.15 | 0.02 |
| P15509 | CSF2RA | Granulocyte-macrophage colony-stimulating factor receptor subunit alpha | 1.04 | 2.06 | 0.04 |
| Q16799 | RTN1 | Reticulon-1 | 1.05 | 2.06 | 0.05 |
| Q9H6X2 | ANTXR1 | Anthrax toxin receptor 1 | 0.93 | 1.91 | 0.05 |
| P25713 | MT3 | Metallothionein-3 | 0.73 | 1.66 | 0.02 |
| O60568 | PLOD3 | Multifunctional procollagen lysine hydroxylase and glycosyltransferase LH3 | -0.64 | 0.64 | 0.02 |
| P07093 | SERPINE2 | Glia-derived nexin | -0.66 | 0.63 | 0.04 |
| Q9BXJ0 | C1QTNF5 | Complement C1q tumor necrosis factor-related protein 5 | -0.69 | 0.62 | 0.02 |
| A0A0B4J1U7 | IGHV6-1 | Immunoglobulin heavy variable 6-1 | -0.80 | 0.57 | 0.03 |
| Q9BTY2 | FUCA2 | Plasma alpha-L-fucosidase | -1.19 | 0.44 | 0.04 |
| O94779 | CNTN5 | Contactin-5 | -2.24 | 0.21 | 0.01 |

**Supplementary Figure 1 shows comparison of ELISA-based quantification of LARGE1 in SMA baseline CSF of pediatric vs adult patients.**


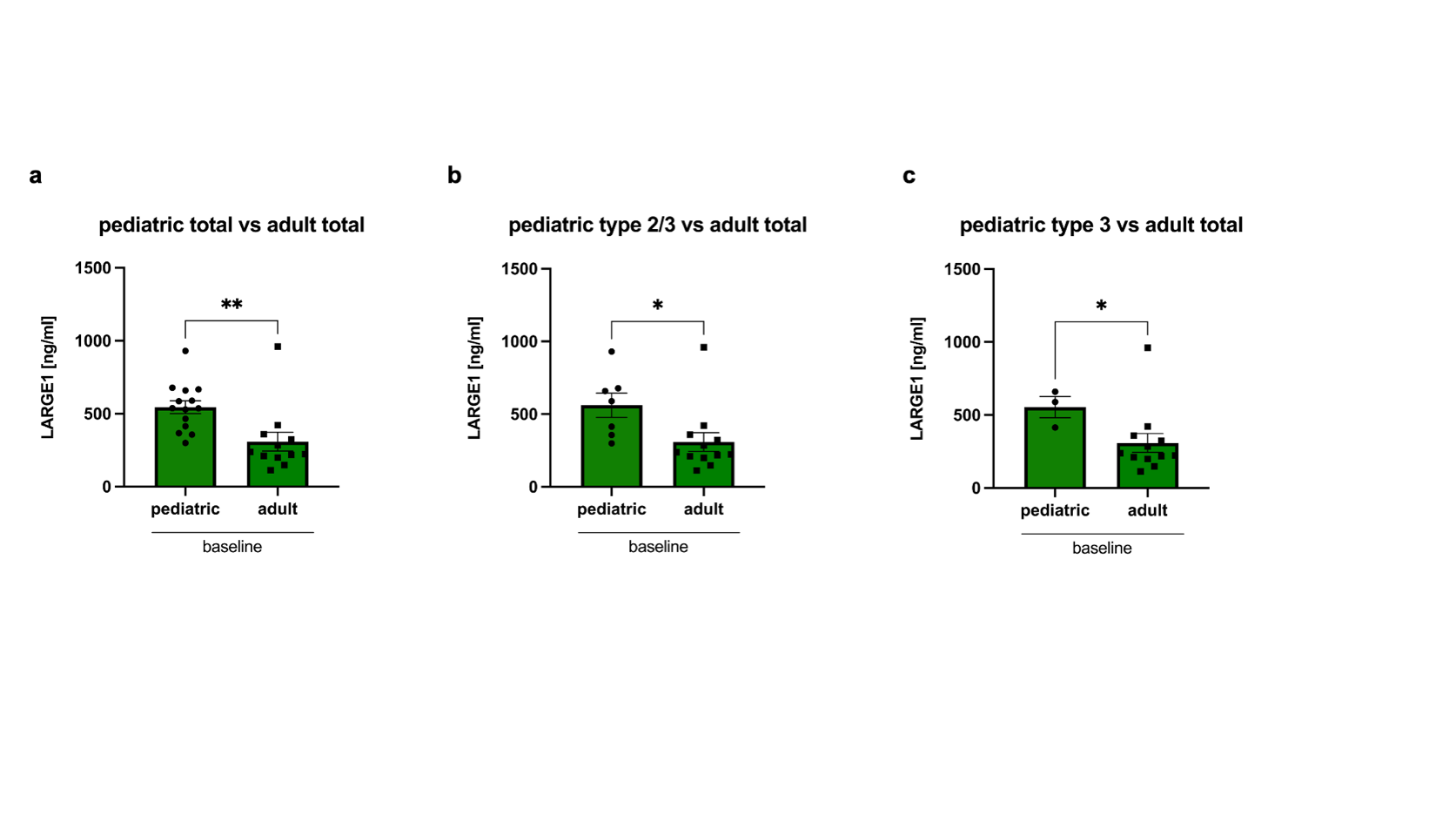


**Supplementary Fig. 1** ELISA-based quantification of LARGE1 in liquor samples derived from 5q-associated pediatric and adult SMA patients. **a,b,c** LARGE1 is significantly increased in pediatric baseline compared to adult baseline in this cohort (p < 0.05; p < 0.01). Abbreviations: ELISA, enzyme-linked immunosorbent Assay; SMA, spinal muscular atrophy.

**Supplementary Figure 2 shows ELISA-based quantification of LARGE1 in disease control CSF (non-inflammatory and inflammatory CNS disease) of pediatric and adult patients**


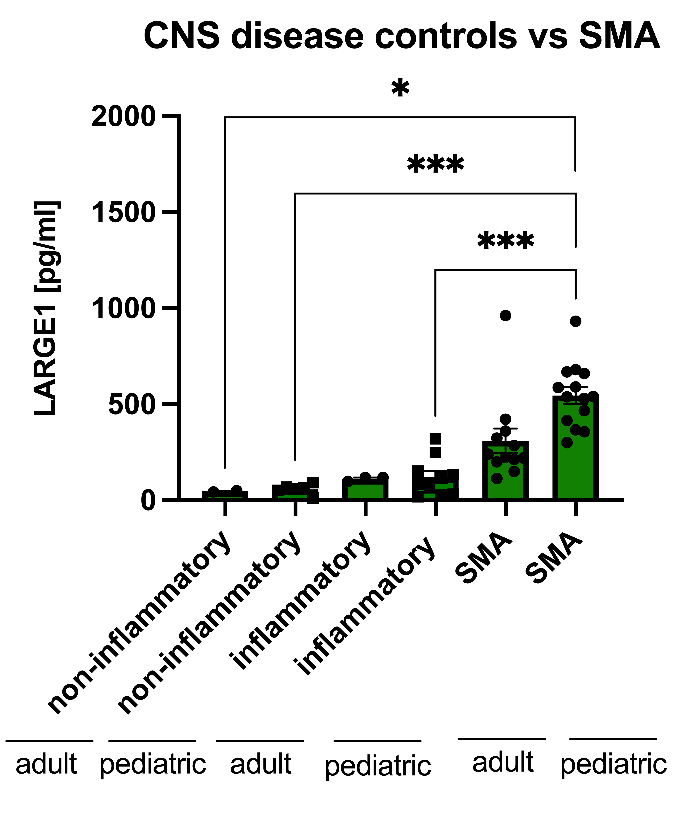


**Supplementary Fig. 2** ELISA-based quantification of LARGE1 in liquor samples derived from CNS disease control as well as 5q-associated SMA patients (adults and pediatric). CNS disease controls are classified into non-inflammatory and inflammatory. Abbreviations: CNS, central nervous system; ELISA, enzyme-linked immunosorbent Assay; SMA, spinal muscular atrophy. p values: *p < 0.05, **p < 0.01, or ***p < 0.001.

**Supplementary Figure 3 shows ELISA-based quantification of LARGE1 in pediatric SMA patients.**


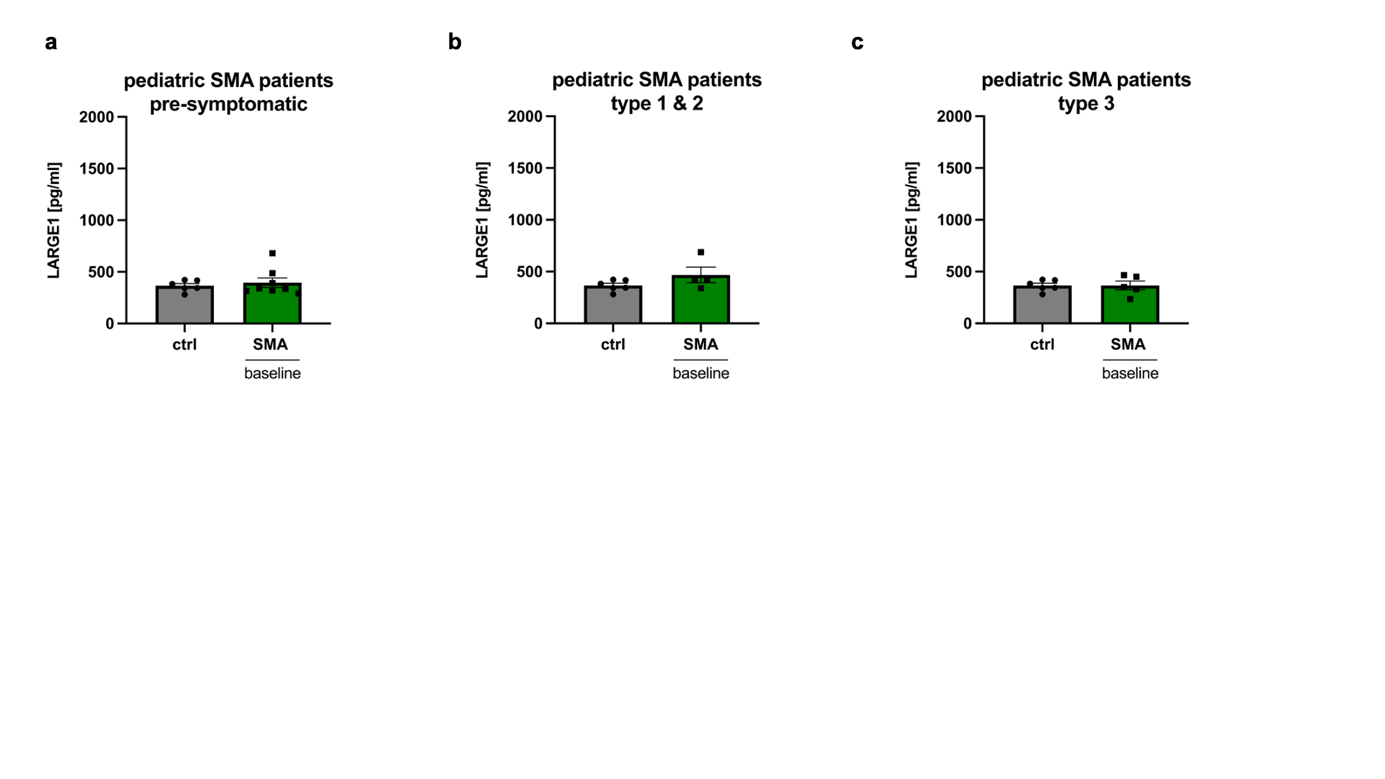


**Supplementary Fig. 3** ELISA-based quantification of LARGE1 in serum samples derived from 5q-associated pediatric SMA patients and controls. **a,b,c** Results obtained in SMA patients with pediatric-onset of disease at baseline visit. No changes of LARGE1 in the total cohort as well as in the subcohort could be measured (p > 0.05). Abbreviations: ctrl, control; ELISA, enzyme-linked immunosorbent Assay; SMA, spinal muscular atrophy.

**Supplementary Figure 4 shows ELISA-based quantification of LARGE1 in pediatric and adult patients of different neuromuscular diseases**


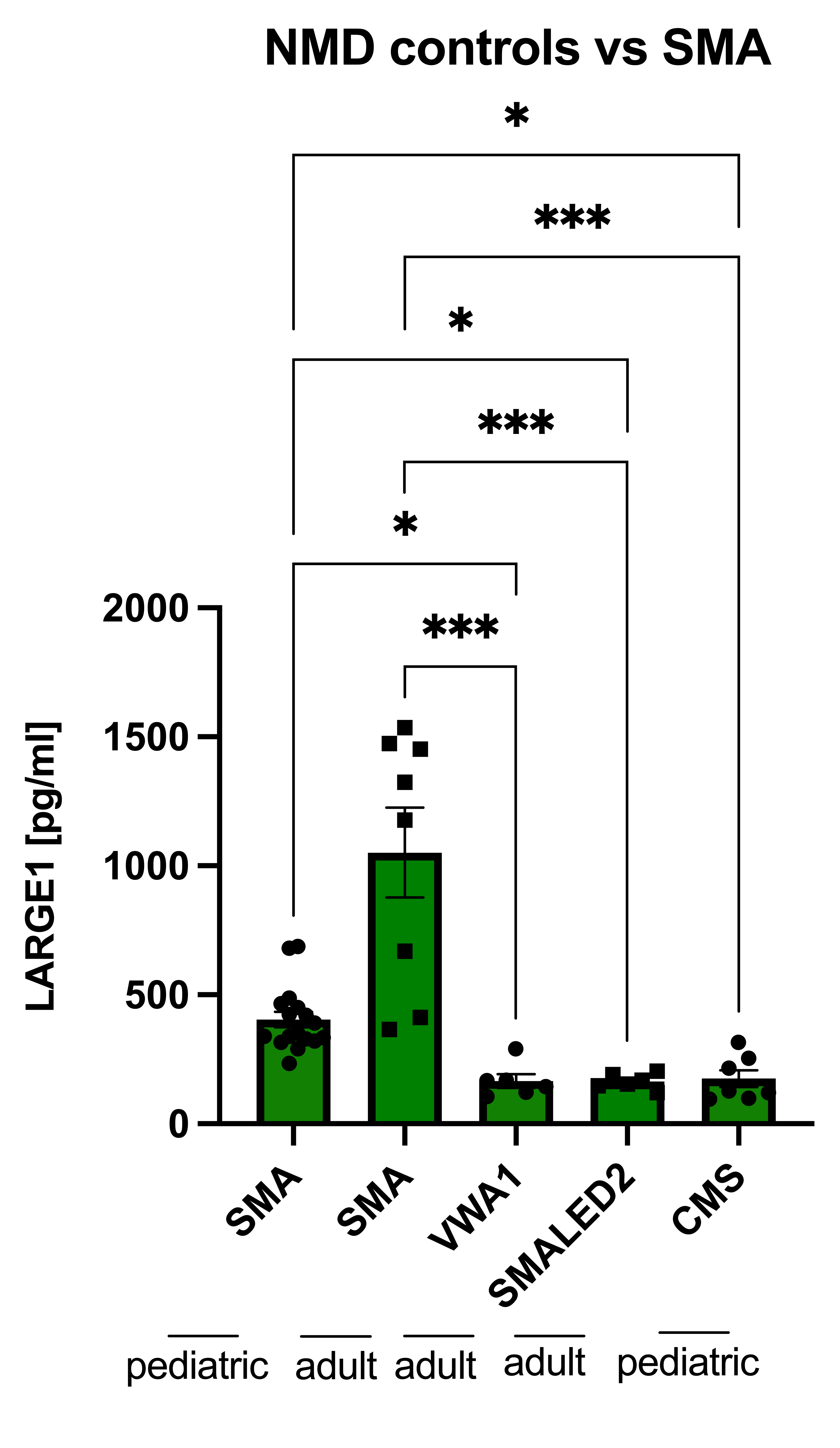


**Supplementary Fig. 4** ELISA-based quantification of LARGE1 in serum samples derived from 5q-associated pediatric and adult SMA patients, adult *VWA1*-patients, adult SMALED2 patients (*BICD2*-related), pediatric CMS patients (*CHRNE*-related). Serum samples of adult and pediatric SMA patients show higher LARGE1 levels compared to other neuromuscular disorders (NMD) (p < 0.05 and p < 0.01). Abbreviations: CMS = congenital myasthenic syndrome; ELISA = enzyme-linked immunosorbent Assay; SMA = spinal muscular atrophy; SMALED2 = autosomal dominant spinal muscular atrophy, lower extremity-predominant type 2; VWA1 = von Willebrand factor A domain containing 1. p values: *p < 0.05, **p < 0.01, or ***p < 0.001.

**Supplementary Figure 5 shows motor neuron staining using SMI-32 at four different time points (P20;P28;P42;P52)**


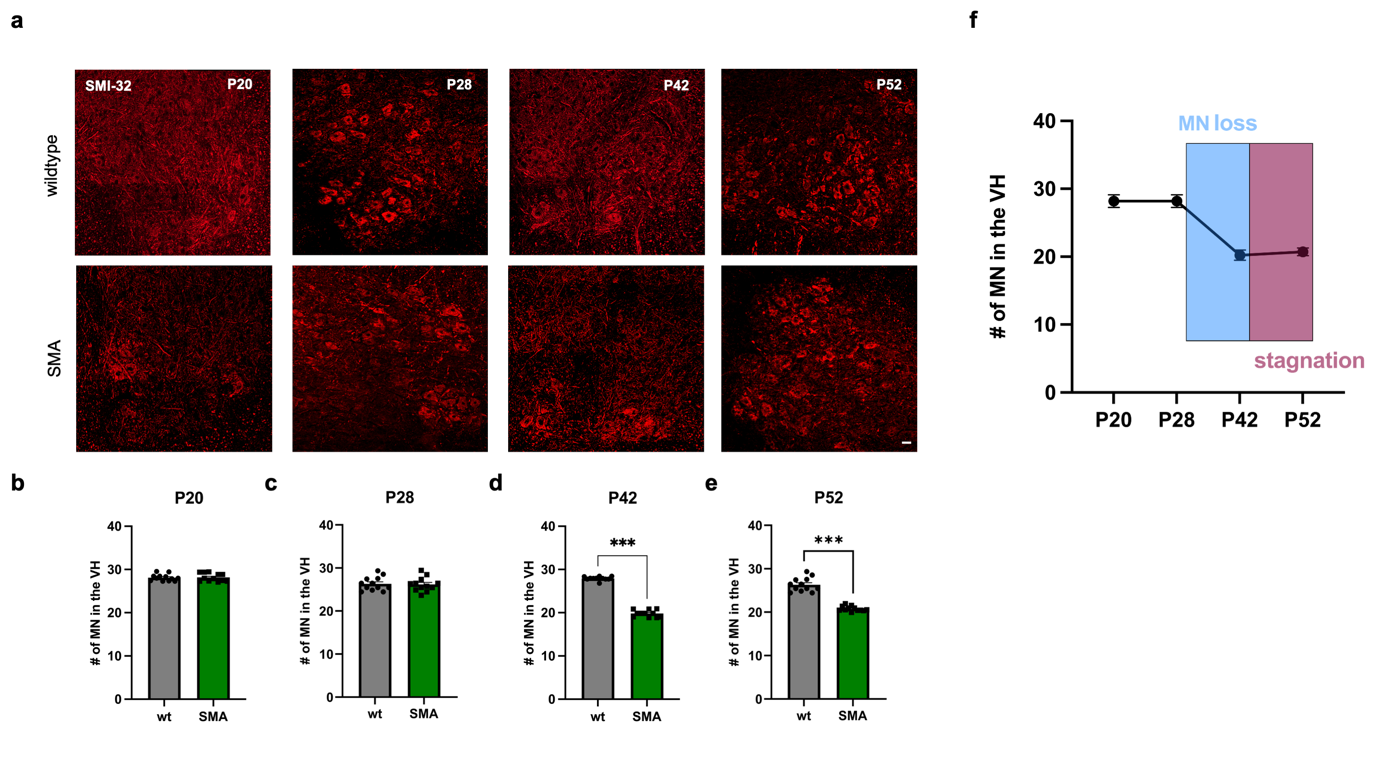


**Supplementary Fig. 5** Mouse model of late-onset SMA shows loss of spinal motor neurons at P52 but not at P28. ***a*** Immunostaining of spinal cord tissue from wild-type (wt) and late-onset SMA mice for motor neuron marker (red) SMI-32 at P28 and P52. Nuclei DNA was stained with DAPI (blue). ***b*** No loss of spinal motor neurons (MN) in SMA mice was observed at P20 (p > 0.05). ***c*** No loss of spinal motor neurons (MN) in SMA mice was observed at P28 (p > 0.05). ***d*** At P42, the number of spinal motor neurons was reduced in SMA mice, compared to wild-type mice at the same age (p < 0.001). ***e*** The number of spinal motor neurons in SMA mice was reduced at P52, compared to wild-type mice at the same age (p < 0.001). ***f*** time course of MN loss divided into an MN loss stage (P28-P42) and a stagnation stage (P42-P52). n = 3 animals per condition (male and female pooled), with 4 slices per animal analyzed (total n = 12 slices per condition). Scale bar: 50 µm. . Abbreviations: MN, motor neuron; SMA, spinal muscular atrophy; P, postnatal day; wt, wild type. p values: *p < 0.05, **p < 0.01, or ***p < 0.001.

**Supplementary Figure 6 shows possible correlation of LARGE1 and MN loss at different time points (P10;P20;P28;P42;P52)**


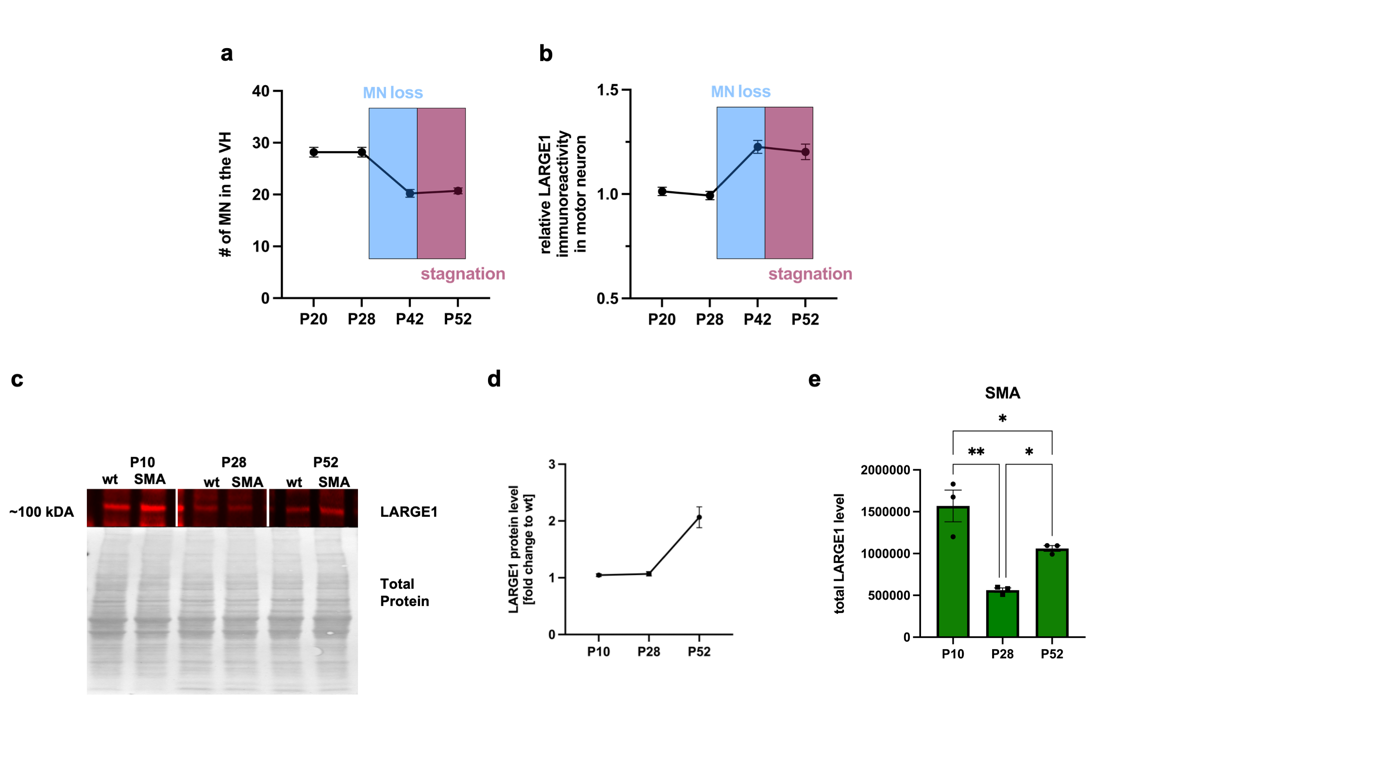


**Supplementary Fig. 6** Correlation of MN loss and LARGE1 expression. ***a*** Mn loss can be classified into two stages, active MN loss (P28-P42) and MN stagnation (P42-P52). ***b*** LARGE1 immunoreactivity over time (P20, P28, P42, P52) with an increase of LARGE1 during the MN loss stage. ***c*** Western blot analysis of LARGE1 in the spinal cord. ***d*** LARGE1 protein level compared to wt mice is increased at P52. ***e*** total level of LARGE1 from SMA mice and WB analysis. P10 is significantly increased compared to P28 and P52 (p < 0.01; p < 0.05) and P52 is increased compared to P28 (p < 0.05). Abbreviations: MN, motor neuron; SMA, spinal muscular atrophy; P, postnatal day; wt, wild type. p values: *p < 0.05, **p < 0.01, or ***p < 0.001.

**Supplementary Figure 7 shows co-localisation of LARGE1 and GM130 at two different time points (P28;P52)**


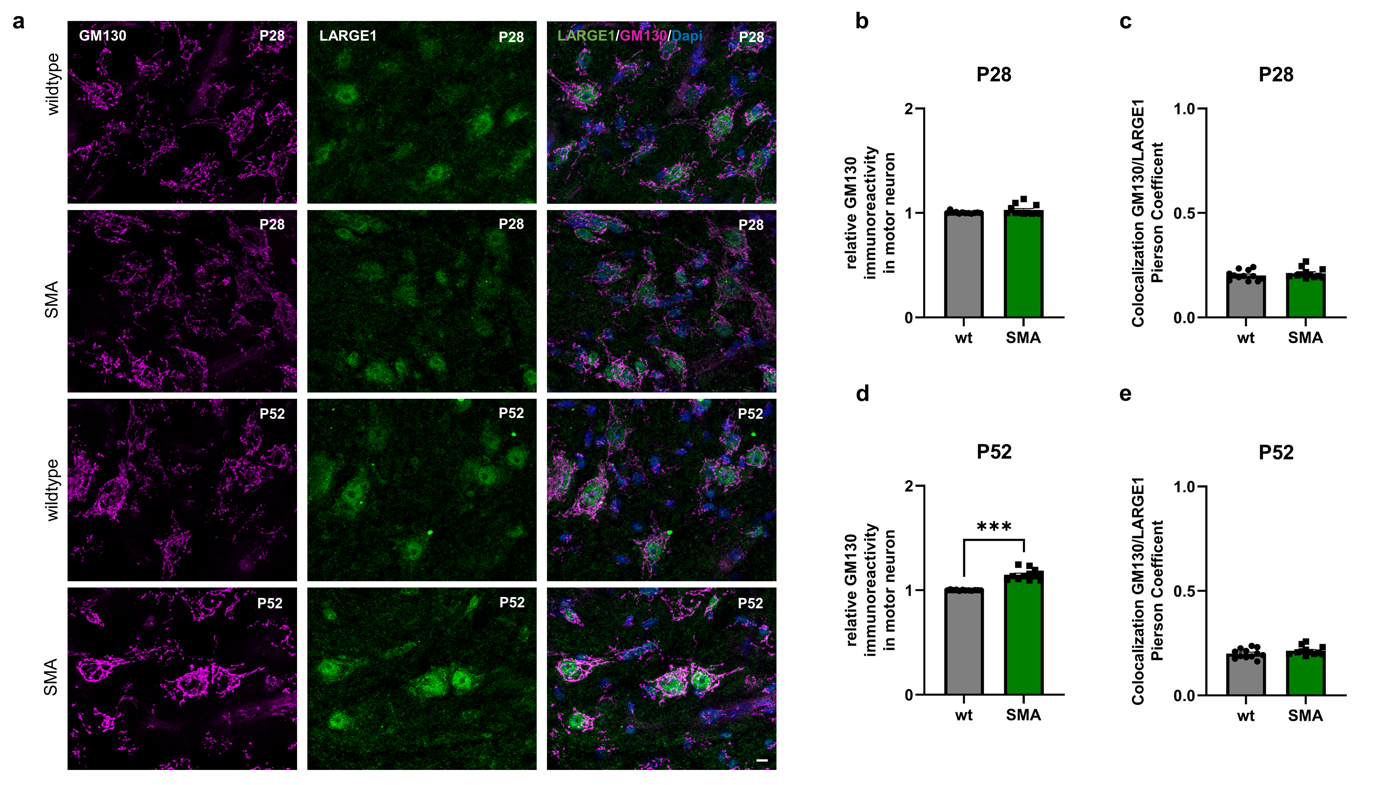


**Supplementary Fig. 7** Expression of Golgi marker GM130 is increased in a mouse model of late-onset SMA at P52 but not at P28. ***a*** Immunostaining of spinal cord tissue from wild-type (wt) and late-onset SMA mice for GM130 (magenta) and LARGE1 (green) at P28 and P52. Nuclei DNA were stained with DAPI (blue). ***b,d*** Immunoreactivity of GM130 was increased in spinal motor neurons of late-onset SMA mice at P52 (p < 0.001) but not at P28 (p > 0.05). ***c,e*** No alteration in GM130/LARGE1 co-expression was observed between wild-type and SMA mice at P28 or P52 (p > 0.05). n = 3 animals per condition (male and female pooled), with 4 slices per animal analyzed (total n = 12 slices per condition). Scale bar: 20 µm. Abbreviations: DNA, deoxyribonucleic acid; SMA, spinal muscular atrophy; P, postnatal day; wt, wild type. p values: *p < 0.05, **p < 0.01, or ***p < 0.001.

**Supplementary Figure 8 shows co-localisation of LARGE1 and BIP at two different time points (P28;P52)**


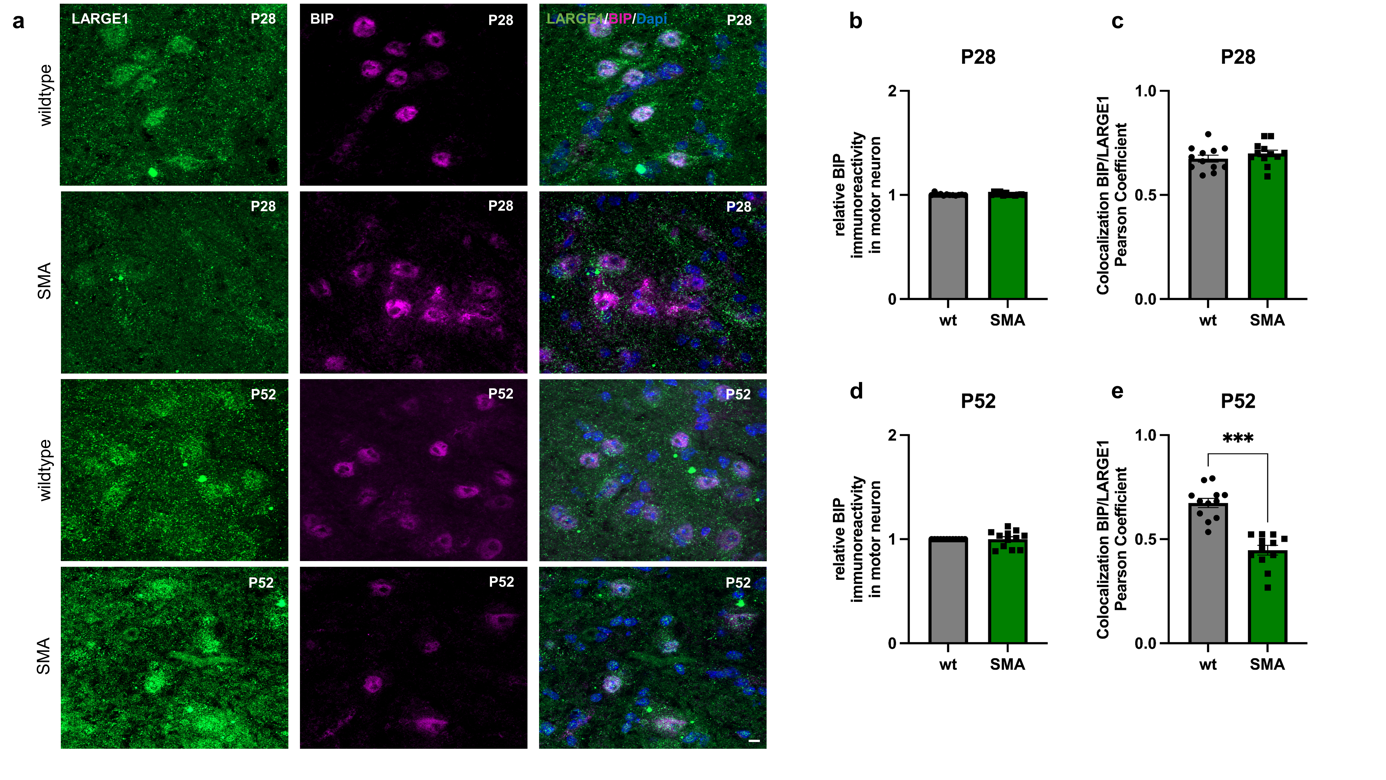


**Supplementary Fig. 8** Co-localization of Endoplasmatic Reticulum marker BiP (major chaperone) and LARGE1 is decreased in a mouse model of late-onset SMA at P52 but not at P28**.** ***a*** Immunostaining of spinal cord tissue from wild-type (wt) and late-onset SMA mice for BIP (magenta) and LARGE1 (green) at P28 and P52. Nuclei DNA was stained with DAPI (blue). ***b,d*** Immunoreactivity of BIP was not affected in spinal motor neurons of late-onset SMA mice at P28 or P52 (p > 0.05). ***c,e*** reduction in BIP/LARGE1 co-expression was observed between wild-type and SMA mice P52 (p < 0.001). n = 3 animals per condition (male and female pooled), with 4 slices per animal analyzed (total n = 12 slices per condition). Scale bar: 20 µm. Abbreviations: DNA, deoxyribonucleic acid; SMA, spinal muscular atrophy; P, postnatal day; wt, wild type. p values: *p < 0.05, **p < 0.01, or ***p < 0.001.

**Supplementary Figure 9 shows spinal cord western blot of wild-type and SMA mice**


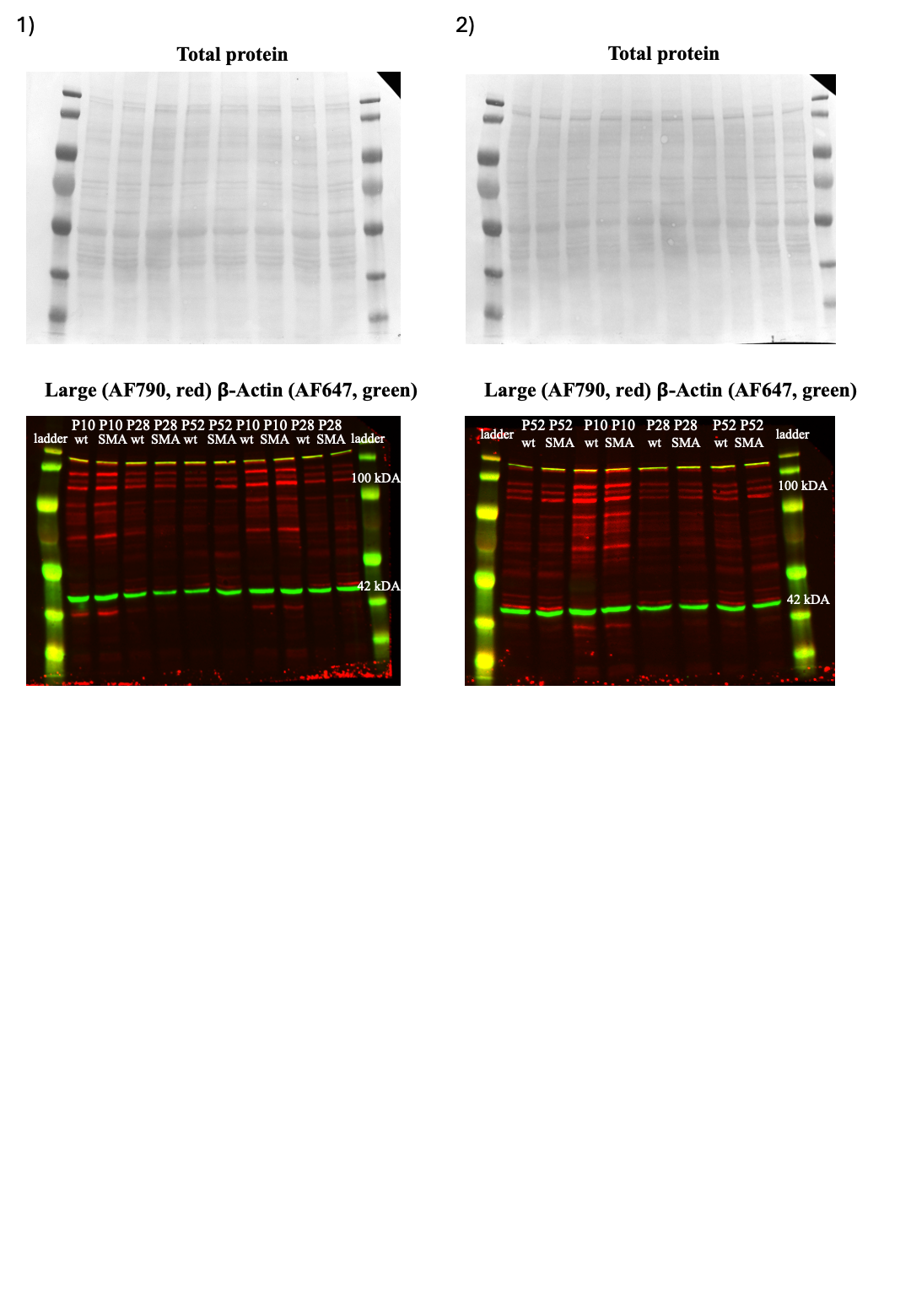


**Supplementary Fig. 9** Total protein staining of the membrane. Western Blot of LARGE1 and Actin of wild-type and late-onset SMA mice at P10, P28 and P52. mice (3 individual mice per condition; P10, P28 and P52; male and female pooled). Total protein, LARGE1 (anti-rabbit, Thermo-Fisher), ß-Actin (anti-mouse, Thermo-Fisher), white box indicates used WB in the manuscript.

**Supplementary Figure 10 shows *tibialis anterior* western blot of wild-type and SMA mice**


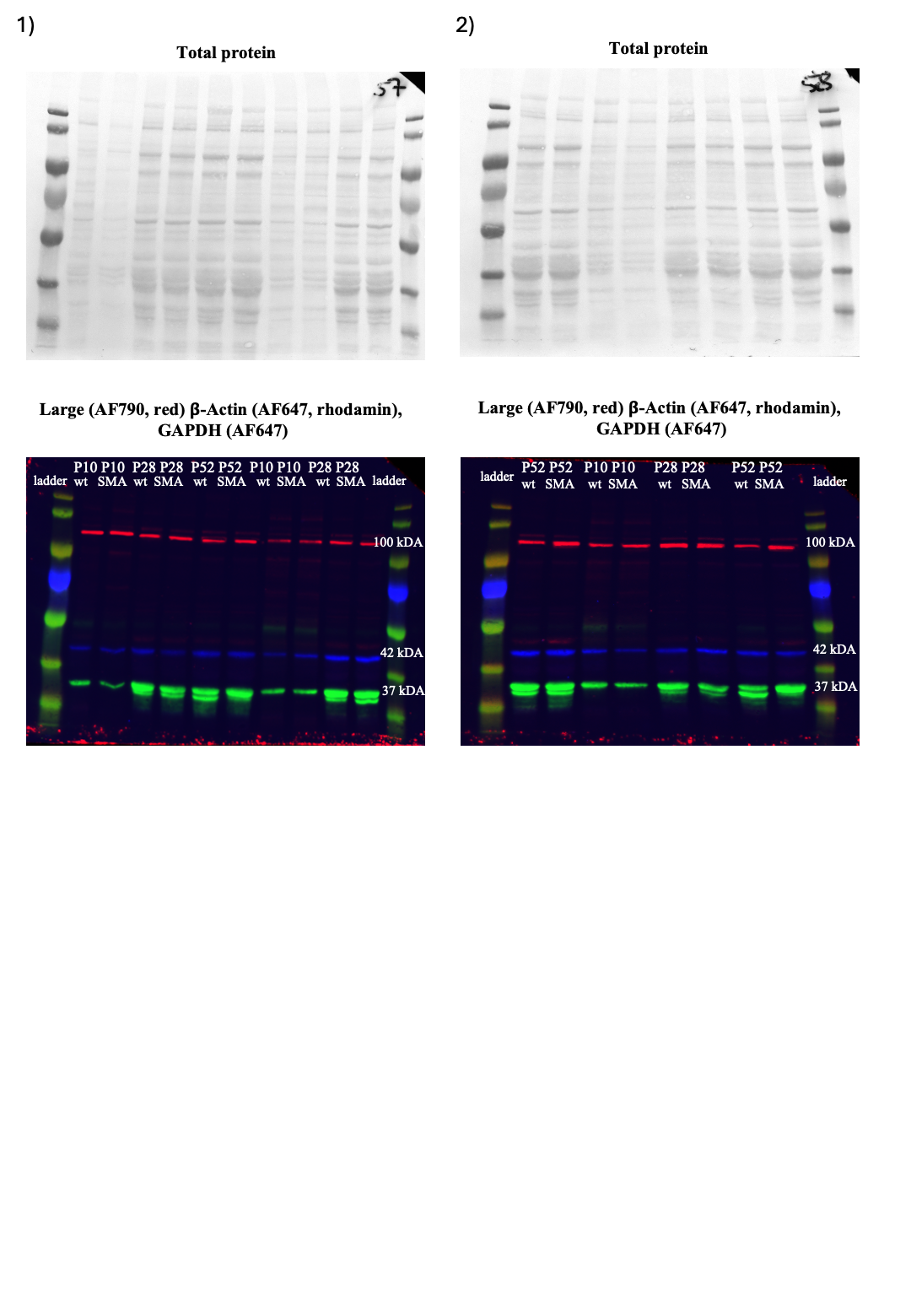


**Supplementary Fig. 10** Total protein staining of the membrane. Western Blot of LARGE1 and Actin of wild-type and late-onset SMA mice at P10, P28 and P52. 3 individual mice per condition; P10, P28 and P52; male and female pooled). Total protein, LARGE1 (anti-rabbit, Thermo-Fisher), ß-Actin (hFAB, BioRad), GAPDH staining (anti-mouse, Thermo-Fisher); white box indicates used WB in the manuscript.
